# Sad from Proteobacteria is a Structurally Distinct ALDH3 Enzyme Specialized for the Oxidation of Steroidal Aldehydes

**DOI:** 10.1021/acs.biochem.5c00213

**Published:** 2025-08-18

**Authors:** Nicolas Rolfe, Dustin Myskiw, Matthew T. Patton, Taylor J. B. Forrester, Matthew S. Kimber, Stephen Y. K. Seah

**Affiliations:** Department of Molecular and Cellular Biology, 3653 University of Guelph, Guelph, Ontario N1G 2W1, Canada

## Abstract

The steroid aldehyde dehydrogenase (Sad) from Proteobacteria is a class 3 aldehyde dehydrogenase (ALDH3) that catalyzes the oxidation of C_3_ steroid side chain aldehydes during bile acid catabolism. The 1.8 Å structure of the enzyme revealed an expanded active site that was able to accommodate bulky steroids, including bile acid intermediates and cholesterol derivatives, with minimal selectivity for ring-conformation or hydroxylation. Sad can utilize both NAD^+^ and NADP^+^ as coenzymes, likely due to a truncated N-terminus and a flexible Glu149 residue, which can avoid steric and electrostatic repulsion with the 2′-phosphate of NADP^+^ while retaining the ability to hydrogen bond to the C2′-OH of NAD^+^. Sad was over 1000-fold more specific for steroid aldehyde substrates than for smaller molecules such as benzaldehyde. Structural comparison with the homologousPseudomonas putida benzaldehyde dehydrogenase (*Pp*BADH) suggested residues that might contribute to the ability of Sad to utilize bulky steroid substrates. Replacement of these residues in an F400A/L125T *Pp*BADH double-variant resulted in a ∼39-fold increase in catalytic efficiency toward steroid aldehyde compared with the wild-type enzyme. This study advances our understanding of the molecular determinants of substrate specificity within the ALDH3 family and lays the groundwork for biocatalytic applications of steroid aldehyde dehydrogenases in the production of steroid pharmaceuticals and the bioremediation of steroidal pollutants.

## Graphical abstract

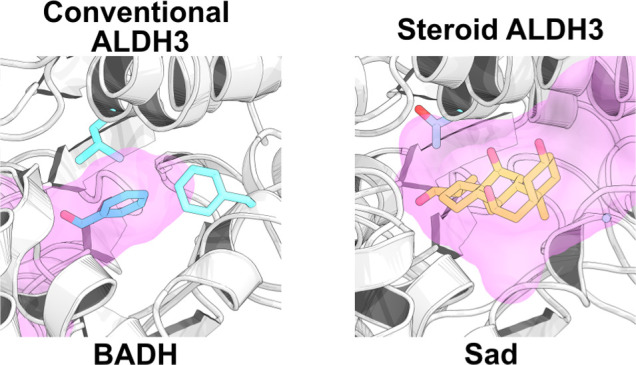

## Introduction

Steroids are isoprene biomolecules characterized by a fused C17 steroid core composed of three six-membered cyclohexane rings (A, B, C) and one five-membered cyclopentane ring (D) (Figure S1).^1^ This rigid core imposes significant steric constraints in conjunction with an extended side chain at the C17 position of the D ring, which introduces additional steric bulk and structural heterogeneity. These features, coupled with limited polar functional groups (e.g., hydroxyl or ketone moieties), render steroids resistant to enzymatic degradation. However, some bacteria have evolved specialized catabolic pathways for steroid utilization. For example, certain Proteobacteria are capable of catabolizing steroidal compounds such as testosterone, estrogen, and bile acids via specialized ring and side chain catabolic pathways.^2^ The steroid side chain degradation reactions share similarities with the canonical fatty acid β-oxidation pathway (Figure S2). The process begins with CoA esterification of the terminal C24 carboxylate, followed by iterative β-oxidation cycles.^3^ Each cycle comprises three sequential enzymatic transformations: (1) α,β-dehydrogenation, (2) double-bond hydration, and (3) aldolytic cleavage (Figure S2).^4,5^ In Proteobacteria, aldolytic side chain cleavage in the first β-oxidation cycle generates acetyl-CoA and a C22 aldehyde intermediate; the latter undergoes further NAD­(P)^+^-dependent oxidation to form a carboxylic acid, priming for a subsequent β-oxidation cycle. Gene knockouts in the Proteobacteria Pseudomonas stutzeri Chol1 implicates Sad in this aldehyde oxidation step (Figures 1 and S2).^5^

**Figure 1:**
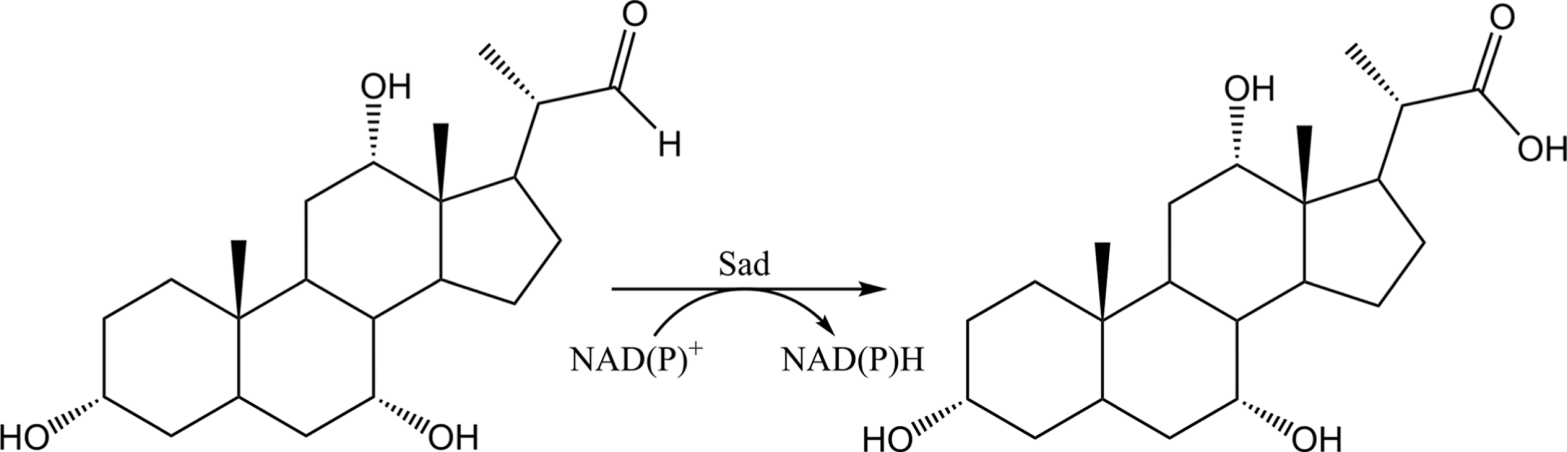
Cholate C_3_ side chain aldehyde oxidation catalyzed by Sad. This reaction requires oxidized nicotinamide cofactor and converts the C22 side chain aldehyde to a C22 carboxylate.

Sad belongs to the ADLH3 family, a phylogenetically distinct clade within the aldehyde dehydrogenase (ALDH) superfamily of NAD­(P)^+^-dependent oxidoreductases.^6,7^ Unlike canonical ALDH enzymes, ALDH3 typically forms dimers rather than tetramers and possesses dual cofactor specificity for both NAD^+^ and NADP^+^, a feature not always observed in other ALDH classes. ALDH3 enzymes also follow a conserved reaction mechanism which involves the following: (1) glutamate-mediated deprotonation of the catalytic cysteine to generate a nucleophilic thiolate; (2) nucleophilic attack on the substrate aldol carbon to form a thiohemiacetal intermediate, a step promoted by a conserved asparagine residue that stabilizes the oxyanion transition state; (3) hydride transfer to NAD­(P)^+^, yielding NAD­(P)H and a thioacyl-enzyme adduct; and (4) hydrolytic cleavage of the thioacyl intermediate to release the carboxylate product.^8,9^ These catalytic residues are conserved in Sad (Figure S3). However, ALDH3 enzymes generally prefer small aromatic aldehydes (e.g., benzaldehyde, coniferyl aldehyde), while Sad uniquely accommodates bulky and hydrophobic steroid aldehydes.^8,9^ This suggests the existance of structural adaptations distinct from the canonical substrate-recognition mechanisms of other ALDH3 enzymes. Here, we report the crystal structure of Sad fromCaldimonas tepidiphilia (Sad_Ctepid_). Coupled with comparative structural, biochemical, and mutagenesis analyses of its homolog, benzaldehyde dehydrogenase (BADH), we elucidated the molecular basis for the differential substrate specificity of these two ALDH3 family members. This work could enable future engineering and use of Sad and other ALDH enzymes to synthesize novel steroidal pharmaceuticals or to remediate harmful steroid pollutants.

## Results

### Cloning, Purification, and Kinetic Activity of Sad

The genes encoding Sad from Comamonas testosteroni KF1 (Sad_Ctest_; RefSeq: WP_043005812.1) and the moderately thermophilic bacterium C. tepidiphilia (RefSeq: WP_119155807.1) were successfully PCR amplified and inserted into Escherichia coli expression vector pET28a. These two proteins share 70% sequence similarity. The N-terminal His-tagged proteins were purified via Ni-NTA affinity chromatography, yielding approximately 9.4 mg of Sad_Ctest_ and 14.2 mg of Sad_Ctepid_ per liter of culture, with molecular weights (assessed by SDS-PAGE) near the theoretical values of 50.9 and 51.3 kDa, respectively (Figure 2A). Both enzymes catalyzed the dehydrogenation of 4-pregnen-3-one-20β-carboxaldehyde, a cholesterol metabolite with a C_3_ side chain aldehyde, in the presence of 1 mM NAD^+^ with similar kinetic parameters (Figure 2B): Sad_Ctest_ had a *K*_m_ of 1.93 ± 0.118 μM and a *k*_cat_ of 3.57 ± 0.0818 s^–1^ (*k*_cat_/*K*_m_ of 1.85 ± 0.459 × 10^6^ M^–1^ s^–1^), while Sad_Ctepid_ had a *K*_m_ of 0.552 ± 0.0315 μM and a *k*_cat_ of 4.90 ± 0.0653 s^–1^ (*k*_cat_/*K*_m_ of 8.88 ± 0.520 × 10^6^ M^–1^ s^–1^) (Figure 2C,D).

**Figure 2:**
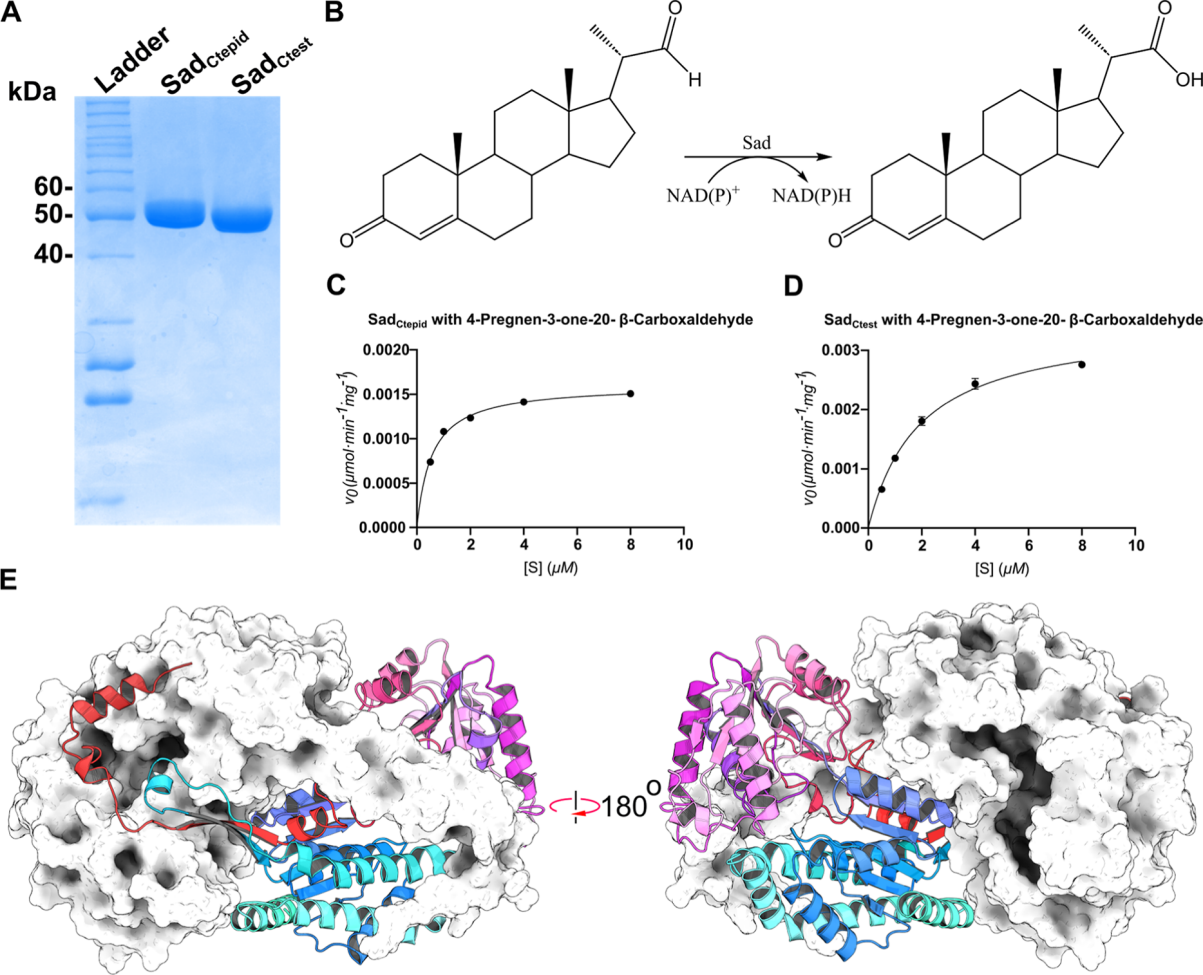
Purification, kinetic activity, and crystallization of Sad. Sad_Ctepid_ and Sad_Ctepid_ were purified via Ni-NTA chromatography and ran on (A) SDS-PAGE with a BenchMark protein ladder. Michaelis–Menten kinetic curves for (B) oxidation of 4-pregnen-3-one-20β-carboxaldehyde as the substrate using (C) Sad_Ctepid_ and (D) Sad_Ctepid_ were measured. Each data point was measured in triplicate. The (E) crystallized structure Sad_Ctepid_ was solved and is shown in opposing views of the protein with one protomer colored from N-terminus to C-terminus in the following order: cyan, blue, purple, pink, then red.

Crystallization trials yielded only needle-shaped crystals for Sad_Ctest_ that were unsuitable for X-ray diffraction analysis. However, Sad_Ctepid_ formed larger, plate-shaped crystals; we therefore targetted this protein for more detailed biochemical characterization.

### Substrate Specificity of Sad_Ctepid_ toward Steroidal and Non-steroidal Aldehydes

Kinetic assays were performed to determine substrate specificity of Sad_Ctepid_ using both steroidal and non-steroidal aldehydes (Table 1). Among steroidal substrates, 4-pregnene-3-one-20β-carboxaldehyde, 3α,7α,12α-trihydroxy-5β-cholan-22-al, and hydroxylated analogs derived from chenodeoxycholate and deoxycholate all showed comparable specificity. Among nonsteroidal aromatic aldehydes, piperonal and benzaldehyde exhibited a 1000-fold lower turnover, while cinnamaldehyde showed a 10,000-fold decrease, and coniferyl aldehyde showed no detectable activity. The small alkyl aldehydes, isobutyraldehyde and propionaldehyde, had turnover 10,000-fold lower than steroids. These results suggest that Sad_Ctepid_ efficiently processes steroidal aldehydes and generally exhibits modest turnover with rigid, aromatic structures bearing C2 side chains, whereas substrates with extended C3 side chains (unbranched) or small, conformationally flexible structures show low turnover.

**Table 1:**
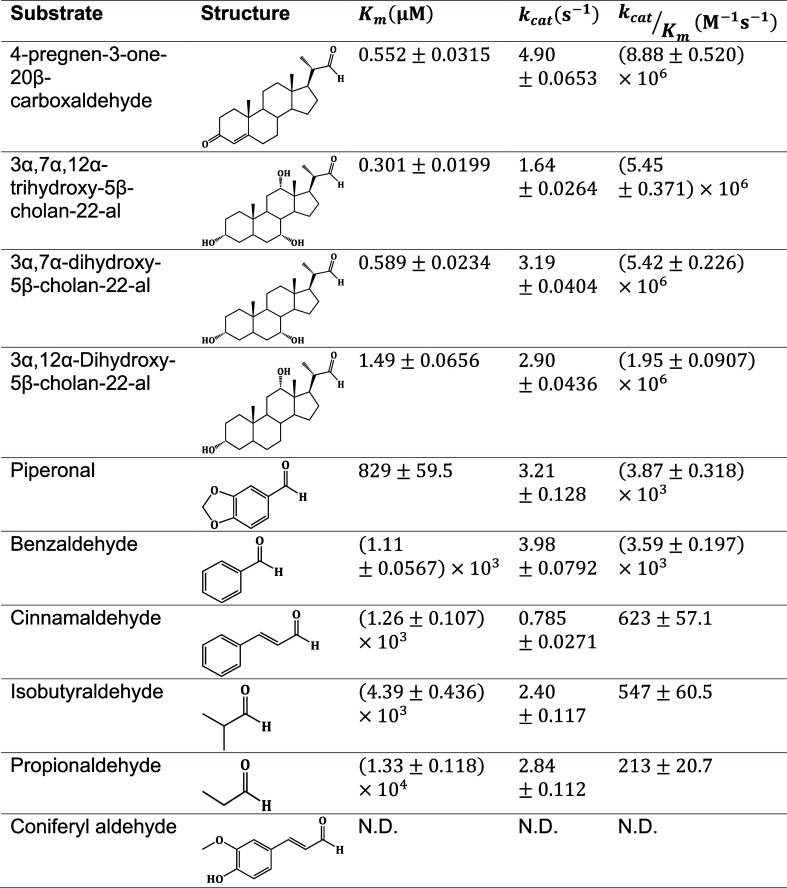
Substrate Specificity Profile for Sad_Ctepid_ a

a All enzymatic reactions were performed in the presence of 1 mM NAD^+^. N.D. indicates not detected; the estimated lower limit of detection corresponds to a *k*_cat_ value <1.34 × 10^–3^ s^–1^.

### Sad_Ctepid_ Catalyzes the Reduction of Steroid Side Chain Carboxylates to Aldehydes

ALDH3 enzymes are typically capable of catalyzing reverse reactions for the reduction of carboxylic acids to aldehydes. We therefore tested reverse reaction catalysis of Sad_Ctepid_ with 4-pregnen-3-one-20β-carboxylic acid, a cholesterol metabolite with a C_3_ side chain and C22 carboxylate group (Figure S5). The reaction was conducted in the presence of 1 mM NADH to facilitate the reductive process, and aldehyde formation was analyzed via mass spectrometry. The aldehyde product, 4-pregnen-3-one-20β-carboxaldehyde (observed *m*/*z* 329), was detected, confirming that the reverse reaction occurred (Figure S5). Kinetic activity of this reaction was low, precluding the accurate determination of Michaelis–Menten parameters. Nevertheless, with 50 μM 4-pregnen-3-one-20β-carboxylic acid and 0.5 mM NADH, the enzyme had a specific activity of 2.94 × 10^–3^ μmol·min^–1^·mg^–1^ in the reverse reaction.

### Sad_Ctepid_ Shows Differential Cofactor Affinity and Catalytic Efficiency for NAD^+^ and NADP^+^

To evaluate cofactor specificity in Sad_Ctepid_, kinetic assays were performed with 4-pregnen-3-one-20β-carboxaldehyde held in excess (60 μM, equivalent to >10× *K*_m_). A *K*_m_ of 69.9 ± 4.14 μM and a *k*_cat_ of 1.20 ± 0.0250 s^–1^ were measured with NAD^+^ as the coenzyme while a much higher *K*_m_ of 628.2 ± 41.2 μM and *k*_cat_ of 4.03 ± 0.120 s^–1^ were obtained with NADP^+^. Overall Sad_Ctepid_ had a slightly higher catalytic efficiency (*k*_cat_/*K*_m_) of about 2.7-fold with NADP^+^ compared NAD^+^ but with a significantly lower (∼9-fold) *K*_m_ for NAD^+^.

### Structure Determination of Sad_Ctepid_

The crystal structure of Sad_Ctepid_ was solved to 1.8 Å resolution by molecular replacement using a protomeric model of the enzyme predicted by ColabFold as the search model (Figure 2E and Table 2).^10^ Like other members of the ALDH3 family, Sad_Ctepid_ is dimeric, with a total interface area of 4685 Å^2^, as measured by PISA.^11^ Sad_Ctepid_ shares structural features with other ALDH3 enzymes: each protomer contains a nucleotide-binding domain with a Rossmann fold (α1−β6), consisting of a five-stranded parallel β-sheet flanked on both sides by α-helices; a catalytic domain (β7−β13) that houses the active site; and an oligomeric arm (α13 and β14) extending from the nucleotide-binding domain of one monomer across the catalytic domain of the adjacent protomer (Figures 2E and S6). The C-terminal α13 on the oligomeric arm, flanked by two 3_10_ helices—one on the β14−α13 loop and one on the α3−β1 loop—encircles the large substrate binding pocket, forming the bulk of the cavity’s entrance. The remainder of the cavity is shaped by the interface between the nucleotide-binding and catalytic domain; the α8−β8, α9−α10, and β13−β14 loops in the catalytic domain, along with the β2−α4 loop and α3 of the Rossmann fold, form the cavity walls. The nucleophilic cysteine, conserved in ALDHs, is positioned on the α8−β8 loop of the catalytic domain.

**Table 2:** Sad_Ctepid_ Data Collection and Refinement Statistics^a^

parameter	statistics
resolution range	44.48–1.8 (1.85–1.8)
space group	*P*2_1_2_1_2_1_
unit cell	82.93 89.45 153.82 90 90 90
total reflections	1,431,732 (111,613)
unique reflections	106,423 (8279)
multiplicity	13.5 (14.0)
completeness (%)	100 (100)
mean I/sigma (I)	11.68 (1.01)
Wilson *B*-factor	24.55
*R*-meas	0.158 (3.174)
CC1/2	0.998 (0.406)
reflections used in refinement	101,512 (9198)
reflections used for *R*-free	1040 (90)
*R*-work	0.1650 (0.3145)
*R*-free	0.1950 (0.3552)
CC (work)	0.959 (0.757)
CC (free)	0.939 (0.638)
number of non-hydrogen atoms	7945
macromolecules	7184
ligands	196
solvent	689
protein residues	940
RMS (bonds) (Å)	0.010
RMS (angles) (deg)	0.91
Ramachandran favored (%)	97.96
Ramachandran allowed (%)	1.83
Ramachandran outliers (%)	0.22
Rotamer outliers (%)	0.82
Clashscore	3.81
average *B*-factor	29.52
macromolecules	28.57
ligands	38.78
solvent	38.44

a Statistics for the highest-resolution shell are shown in parentheses.

### Sad_Ctepid_ Utilizes an Expanded Active-Site Cavity for Steroid Binding

A DALI search of the Protein Data Bank shows that the closest structural homologues of Sad_Ctepid_ are other ALDH3 enzymes with pairwise RMSD values ranging from 1.8 to 2.1 Å. Representative examples include the xenobiotic-detoxifying ALDH3 enzymes from Homo sapiens (*Hs*ALDH; PDB 8BB8) and Rattus norvegicus (PDB 1AD3), benzaldehyde dehydrogenase from Pseudomonas putida (*Pp*BADH; PDB 5UCD), and 4,4′-diaponeurosporen-aldehyde dehydrogenase from Staphylococcus aureus (*Sa*ALDH; PDB 6K0Z), an ALDH3 involved in staphyloxanthin synthesis.^9,12–14^

Structural comparisons of the binding cavity architecture of Sad_Ctepid_ with its closest structural homologues suggests that this site is adapted for steroid binding through a combination of backbone remodelling and reductions in bulk of cavity-lining residues. Sad_Ctepid_ has a wide and deep active site pocket with a volume of ∼1133 Å^3^. For reference, the solvent-accessible volume of 3α,7α,12α-trihydroxy-5β-cholan-22-al (Table 1) is ∼530 Å^3^ (calculated using MoloVol).^15^

The active site of Sad_Ctepid_ is larger than its physiological steroid substrate and significantly larger than that of any of its previously studied homologues. Human and rat ALDH3s have active site cavities that form shallow grooves (727–748 Å^3^), while bacterial ALDH3s like *Pp*BADH (239 Å^3^) and *Sa*ALDH (131 Å^3^) have substrate binding sites that are relatively narrow and shallow, and, in the case of *Sa*ALDH, wholly closed off to the solvent. In Sad_Ctepid_, the N-terminal end of the α3 helix incorporates a single π-helix turn but is otherwise relatively straight; this leaves an open space between itself and the α8−α9 loop that is part of the active site pocket (Figure 3A). In *Pp*BADH, α3 is shifted inward through a pair of π-bulges, where it packs against a short helix inserted into the equivalent loop; this closes this space, leaving only an adjacent narrow tunnel (Figure 3B). Other ALDH3 enzymes have an intermediate structure and leave a narrower gap.

**Figure 3:**
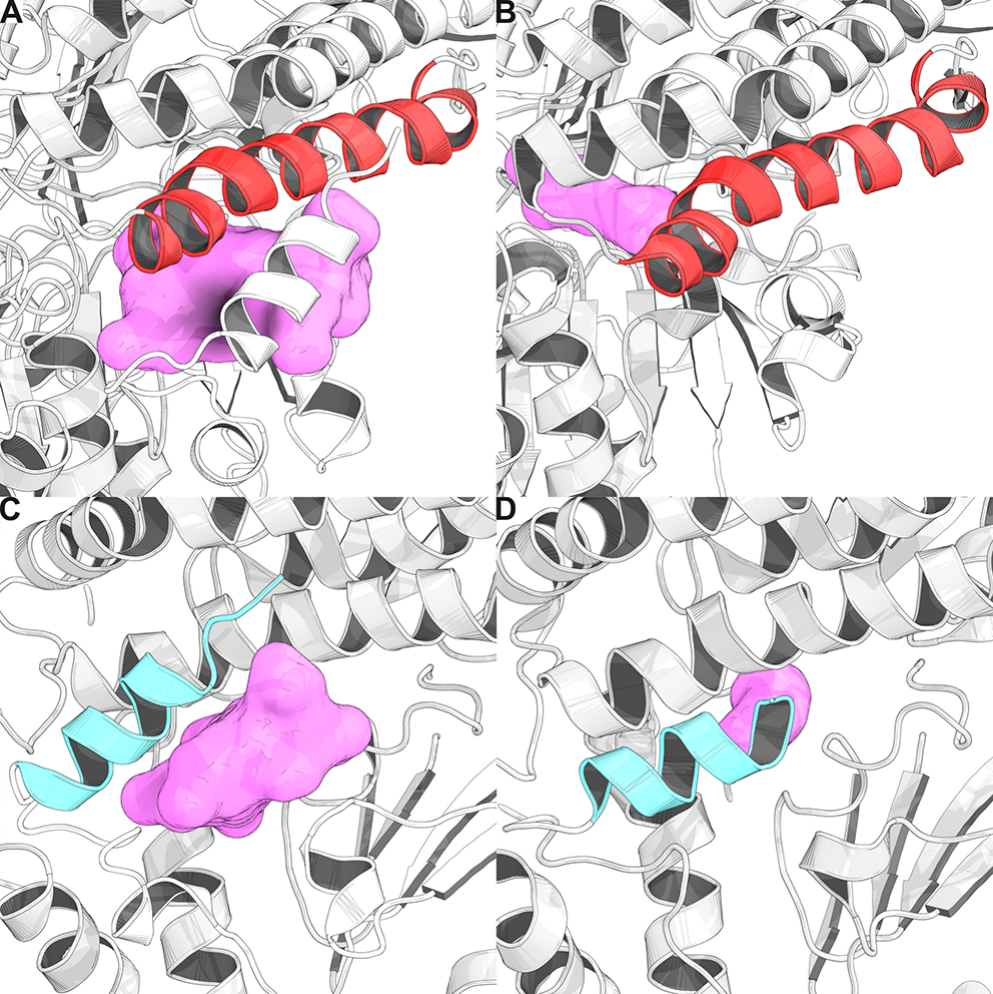
Structural comparison of Sad_Ctepid_ with bacterial homologues *Pp*BADH and *Sa*ALDH. The (A) α3 helix (red) in Sad_Ctepid_ adopts a less pronounced kink compared to (B) *Pp*BADH, elevating its position and enlarging the binding cavity (shown as a indigo-colored surface view) by reducing steric bulk. In Sad_Ctepid_, the (C) α13 helix (cyan) is angled away from the active site entrance, contrasting with (D) *Sa*ALDH, where its displacement partially occludes the cavity. These structural differences suggest adaptations in Sad_Ctepid_ that facilitate steroid binding.

The C-terminal oligomeric arm of the adjacent protomer can also contribute to the active site: in *Pp*BADH, this region is truncated, resulting in a shallow pocket much closer to the surface; in *Sa*ALDH, the α13 helix is mobile and shifts over the active site tunnel, closing it off from bulk solvent (Figure 3D).^9,14^ In Sad_Ctepid_ and *Hs*ALDH3, the oligomeric arm circles around the active site pocket, building up tall walls on three sides that contribute to its much greater depth (Figure 3C). In addition, an alignment of the residues lining the *Pp*BADH substrate binding cavity with Sad_Ctepid_ reveal a pattern where bulky residues are consistently replaced with smaller residues (Figure 4). These changes include His74 → Ala75, Glu75 → Ser76, Tyr121 → Ala124, Leu125 → Thr128, Ile250 → Val128, and Phe400 → Ala412. Additionally, a 3_10‑_helix on the β13−β14 loop is shorter in Sad_Ctepid_ than that in *Pp*BADH, which appears to be a result of A399 in *Pp*BADH substituted with P399 in Sad_Ctepid_, disrupting the helix (Figure 4).

**Figure 4:**
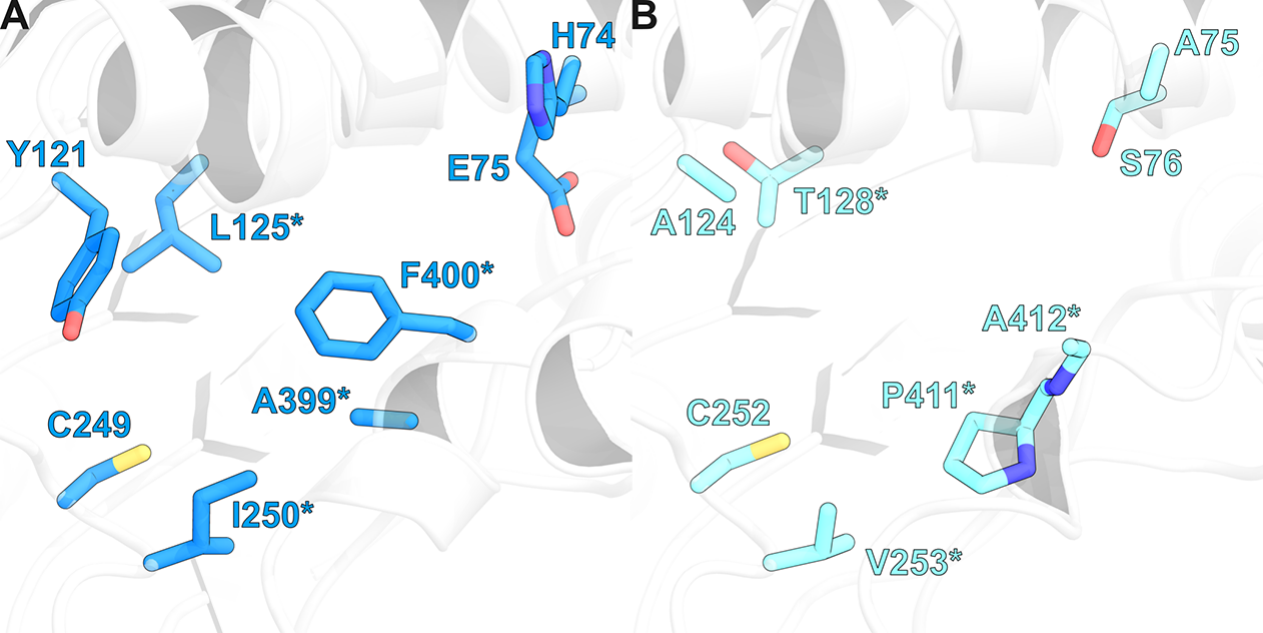
Comparison of residues surrounding the binding pockets of *Pp*BADH and Sad_Ctepid_. Multiple bulky residues in (A) *Pp*BADH: Ile250, His74, Glu75, Leu125, Phe400, and Tyr121 are replaced with less bulky residues in (B) Sad_Ctepid_: Val253, Ala75, Ser76, Thr128, Ala412, and Ala124, respectively, enlarging the binding cavity. Asterisks indicate residues that enhanced steroid activity in *Pp*BADH when substituted with the structurally aligned residues from Sad_Ctepid_. Residues without an asterisk did not improve steroid catalysis. The catalytic cysteine (Cys249 in *Pp*BADH and Cys252 in Sad_Ctepid_) is shown as a positional reference to indicate the approximate location where the aldehyde group of the steroid side chain would be located.

### Engineering *Pp*BADH for Steroid Catalysis

To investigate the roles of the residues mentioned above in steroid binding, we performed site-specific mutagenesis in *Pp*BADH, replacing residues with those found in Sad_Ctepid_. The melting temperatures (*T*_m_) of these variants, determined by thermal shift assays, were generally similar to the wild-type enzyme, indicating minimal disruption of the overall enzyme structures (Table S1). Wild-type *Pp*BADH had very low activity toward 4-pregnene-3-one-20β-carboxaldehyde which precluded accurate determination of kinetic parameters; therefore, specific activity was determined (Table 3).

**Table 3:** Apparent Specific Activity (SA) Measurements for *Pp*BADH and Variants with 50 μM 4-Pregnene-3-one-20β-carboxaldehyde and 1 mM NAD^+^

*Pp*BADH variant	SA (μmol·min^–1^·mg^–1^)
wt	3.32 × 10^–4^
E75S	1.03 × 10^–4^
Y121A	1.53 × 10^–4^
H74A	1.63 × 10^–4^
A399P	6.69 × 10^–4^
I250V	7.79 × 10^–4^
F400A	1.55 × 10^–3^
L125T	7.71 × 10^–3^
L125T/F400A	1.29 × 10^–2^

The A399P and I250V substitutions increased steroid activity by approximately 2-fold. The A399P mutant was initially included to disrupt the β13–β14 3_10_-helix similar to P411 in BADH, potentially reducing steric bulk near the steroid A and B rings. However, the activity was only increased marginally. An alternative explanation may be that the steroid rings pack more optimally against proline. The E75S, Y121A, and H74A substitutions resulted in about a 2-fold decrease in activity (Table 3).

The L125T and F400A variants exhibited approximately 24-fold and 5-fold higher activity toward the steroid substrate than the wild-type. This enabled the determination of kinetic parameters for these variants (Table 4). A double-variant with both L125T and F400A replacements further increased activity (39-fold), with lower *K*_m_ and higher *k*_cat_ values compared to the single variants (Table 4). We also tested these variants with benzaldehyde (Table 5). The I250V substitution exhibited a 7-fold increase in *k*_cat_ but also an approximately 1.5-fold increase in *K*_m_ for benzaldehyde. The L125T substitution had a 2-fold lower *K*_m_ and a 9-fold lower *k*_cat_.

**Table 4:** Apparent Steady-State Kinetic Parameters for *Pp*BADH L125T and F400A Variants with 4-Pregnene-3-one-20β-carboxaldehyde and 1 mM NAD^+^

*Pp*BADH variant	*K*_m_ (μM)	*k*_cat_ (s^–1^)	$k_{cat}/K_{m}(M^{-1}\;s^{-1})$
F400A	4.35 ± 0.167	(2.84 ± 0.0334) × 10^–3^	653 ± 75.1
L125T	18.94 ± 1.07	(2.91 ± 0.0667) × 10^–2^	1540 ± 36.8
L125T/F400A	1.73 ± 0.0801	(4.31 ± 0.0719) × 10^–2^	(2.49 ± 0.123) × 10^4^

**Table 5:** Apparent Steady-State Kinetic Parameters for *Pp*BADH and Variants with Benzaldehyde and 1 mM NAD^+^

*Pp*BADH variant	*K*_m_ (μM)	*k*_cat_ (s^–1^)	kcatKm(M−1s−1)
Wt	5.99 ± 0.363	1.18 ± 0.0246	(1.97 ± 0.126) × 10^5^
E75S	19.8 ± 1.49	0.931 ± 0.0225	(4.70 ± 1.19) × 10^4^
Y121A	700.4 ± 31.9	0.867 ± 0.0137	(1.24 ± 0.0598) × 10^3^
H74A	5.81 ± 0.412	1.42 ± 0.0263	(2.44 ± 0.179) × 10^5^
A399P	8.58 ± 0.505	0.0161 ± 0.000153	(1.88 ± 0.112) × 10^3^
I250V	8.86 ± 0.770	8.14 ± 0.277	(9.19 ± 0.858) × 10^5^
F400A	59.5 ± 2.59	0.469 ± 0.00623	(7.88 ± 0.359) × 10^3^
L125T	3.18 ± 0.216	0.131 ± 0.00312	(4.12 ± 0.297) × 10^4^
L125T/F400A	40.97 ± 2.58	0.0370 ± 0.000756	903 ± 59.8

In summary, these substitutions decreased *k*_cat_/*K*_m_ with benzaldehyde by up to approximately 160-fold, while concomitantly improving the ability to accommodate the bulkier steroid substrate. The *k*_cat_/*K*_m_ for the double-variant is only about 8-fold lower than that of wild-type *Pp*BADH with its physiological substrate, benzaldehyde; it is worth noting, however, that the *k*_cat_ for the steroid substrate remains 2 orders of magnitude lower than that for benzaldehyde, suggesting that although widening the active site allows this bulky molecule better access to the catalytic machinery, positioning of the steroid substrate for optimal catalysis requires additional changes in the active site.

As attempts to capture an enzyme–substrate complex by X-ray crystallography were unsuccessful, we manually modeled 3α,7α,12α-trihydroxy-5β-cholan-22-al (Table 1) into the active site (Figure 5). The rigid steroid nucleus was positioned within the binding pocket of Sad_Ctepid_ to fit the available space, while the aldehyde was positioned to form productive interactions with the catalytic residues. This model was then energy minimized using Rosetta3.^16^ In the minimized binding pose, the hydrophobic steroid rings form van der Waals interactions with nonpolar residues lining the cavity walls, while the hydrophilic face remains exposed in an open space, surrounded by polar regions where water molecules could potentially bridge them. One of the ring hydroxyl groups, C7-OH, forms a direct hydrogen bond with the side chain amide of Asn68; this residue could also potentially rotate to a hydrogen bond with C12-OH, which is positioned in an open pocket large enough to also accommodate water, while C3-OH is exposed to bulk solvent. The aldehyde is positioned adjacent to Cys252, with its oxygen atom placed within the oxyanion hole formed by the Asn123 side chain Nδ and the Cys252 backbone amide nitrogen atom. The model suggests that substituting Ala412 with Phe400 from *Pp*BADH would result in steric clashes with the A and B rings due to the phenyl ring positioning, while replacing Thr128 with Leu125 would clash with C21 of the side chain methyl branch, assuming the side chains adopt similar conformations as in *Pp*BADH (Figure 5).

**Figure 5:**
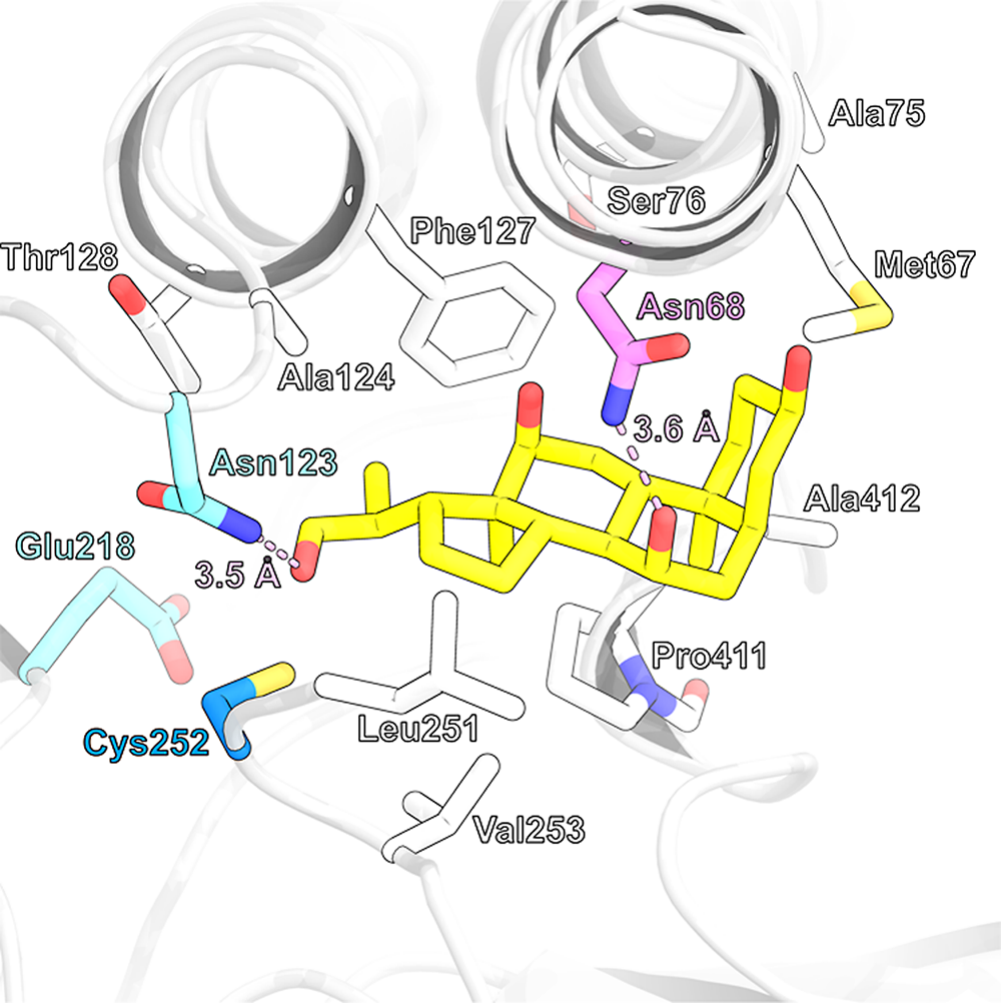
Molecular modeling of 3α,7α,12α-trihydroxy-5β-cholan-22-al in the active site of Sad_Ctepid._ The modeled substrate is shown in yellow. The catalytic cysteine is shown in light blue, with other residues involved in catalysis depicted in cyan. Hydrogen bonding is represented as pink dashed lines. Asn68, highlighted in violet, forms a hydrogen bond with the C7-OH group, while Asn123 hydrogen bonds with the side chain aldehyde. Residues that do not participate in hydrogen bonding but contribute to steric packing of the steroid nucleus or equivalent residues replaced in *Pp*BADH are shown as white sticks.

## Discussion

In the first β-oxidation cycle of bile acid side chain catabolism in Proteobacteria, Sad oxidizes the C22-aldehyde of C_3_ steroid side chains to a carboxylate. Our findings show that purified Sad_Ctest_ and Sad_Ctepid_ homologues had comparably high activity toward steroid substrates. The structure of Sad_Ctepid_, supported by mutagenesis and biochemical data, revealed an enlarged substrate binding site relative to the homologous ALDH3 members (Figure 3). This structural adaptation may underlie the broad substrate specificity of this enzyme. Sad_Ctepid_ had similar activity toward a range of bile acid metabolites, such as deoxycholate and chenodeoxycholate, where variations in steroid core hydroxylation appear to have a minimal effect on the catalytic efficiency (Table 1). Molecular modeling of 3α,7α,12α-trihydroxy-5β-cholan-22-al in the active site (Figure 5) suggests that a direct hydrogen bond forms between C7-OH and Asn68, consistent with 3α,12α-dihydroxy-5β-cholan-24-al, a deoxycholate aldehyde intermediate lacking the C7-OH, exhibiting slightly reduced (2.8-fold) catalytic efficiency compared to bile acids with a C7-OH group. Additionally, Sad_Ctepid_ demonstrated comparable activity with 4-pregnen-3-one-20β-carboxaldehyde, a cholesterol intermediate with an unsaturated A-ring (Table 1). In cholesterol derivatives, the A ring is unsaturated and planar, in contrast to that in bile acids, where it is orthogonal to the other rings. The distal region of the active site cavity is shaped so to allow the A ring to pack against a nonpolar surface in either configuration (Figures 5 and S7).

In Sad_Ctepid_, the determinants of steroid binding appear to stem from local remodelling of the protein backbone to widen the entrance and innermost regions of the binding pocket. The substitution of bulkier residues in the catalytic site of homologues with smaller residues also increases binding cavity volume. In tandem, these unique structural features enable Sad_Ctepid_ to accommodate steroids in contrast to other ALDH3 enzymes. We have shown that *Pp*BADH, which normally oxidizes benzaldehyde, can be engineered to accept steroids by applying analogous widening substitutions. Despite this, the best variants show a *K*_m_ for steroids lower than wild-type *Pp*BADH exhibits for benzaldehyde; however, *k*_cat_ remains 2 orders of magnitude lower. This likely reflects an inefficient organization of the substrate in the active site. Although the modified pocket accommodates the larger substrate, the low *k*_cat_ suggests that productive complexes form infrequently. Turnover may require sampling a higher-energy, strained conformation that is accessible within the protein’s internal dynamics but is sampled less frequently than the catalytically productive state in wild-type *Pp*BADH, leading to a reduced *k*_cat_.

Our mutagenesis studies revealed a clear trade-off between catalytic efficiency and structural stability in *Pp*BADH variants. Specifically, the L125T variant had the largest change in melting temperature compared to the wild-type (Δ*T*_m_ – 4.1 °C) among all tested substitutions (Table S1), yet displayed significant improvement in the catalytic efficiency (∼24-fold) toward steroid substrates (Table 4). The double variant L125T/F400A similarly showed reduced thermal stability (Δ*T*_m_ – 3.4 °C) while maintaining the highest activity. These results are consistent with the common phenomenon in protein engineering where mutations that expand substrate scope or increase catalytic activity can compromise protein stability, likely by introducing local flexibility or disrupting stabilizing interactions of the targeted residues. The extent of thermal destabilization observed here is modest, and both variants retained robust activity under assay conditions, suggesting that their functional stability remains sufficient for effective catalysis. Nevertheless, this balance between enhanced activity and diminished stability is a potential limitation for applications requiring prolonged enzyme function or exposure to elevated temperatures. Future work could address this by introducing compensatory stabilizing mutations, either through rational design or directed evolution, to restore or enhance thermal resilience without sacrificing the improved catalytic properties. Additionally, the strong synergy observed in the L125T/F400A double mutant suggests that combining mutations may yield more than additive effects. Variants such as E75S, Y121A, or H74A, which showed limited improvement when tested individually, may still contribute positively when paired with productive substitutions. Exploring such combinations could reveal new variant classes with both high activity and improved stability, expanding the practical utility of engineered *Pp*BADH for biocatalysis involving steroid substrates.

Sad_Ctepid_ demonstrated a preference for NAD^+^ over NADP^+^, indicated by a ∼9-fold lower *K*_m_ for NAD^+^. This is consistent with ALDH3 enzymes, which typically accommodate both cofactors but favor NAD^+^, while enzymes from other ALDH families are generally specific for a single cofactor. A comprehensive comparison of numerous ALDH enzymes suggest that Glu149 (corresponding to Glu195 in ALDH2 fromH. sapiens), located on the β3−α5 loop of the nucleotide-binding domain, plays a key role in cofactor discrimination by coordinating the adenine ribose 2′-OH in NAD^+^ while avoiding steric clashes with the 2′-phosphate of NADP^+^.^17^ The inclusion of Gly177 (equivalent to Tyr244 in ALDH2 from H. sapiens) on the β4−α6 loop enables conformational flexibility of Glu149, allowing it to shift away from the 2′-phosphate group in NADP^+^.^17^ The truncated N-terminus of ALDH3 enzymes, including Sad_Ctepid_, which lacks ∼60 residues present in *Hs*ALDH2, creates additional space around Glu149, further reducing steric constraints.^17^

Sad_Ctepid_ can oxidize several nonsteroidal aldehydes, albeit with reduced catalytic efficiency (Table 1). Aromatic aldehydes with C_2_ side chains, such as piperonal and benzaldehyde, show moderate *k*_cat_/*K*_m_ values, though these are 3 orders of magnitude lower than those for steroids. The reduced turnover suggests that the alternate ring structure of these substrates has less optimal binding interactions compared to steroid nuclei within the substrate-binding pocket. In contrast, aliphatic aldehydes likely bind too loosely within the pocket for the proper positioning of the aldehyde group. Cinnamaldehyde showed significantly lower activity than benzaldehyde, suggesting that its extended unsaturated side chain is not optimally positioned within the active site. Interestingly, Sad_Ctepid_ is annotated as a coniferyl aldehyde dehydrogenase (WP_119155807.1) but shows no detectable activity with coniferyl aldehyde, possibly due to its extended unsaturated side chain in addition of functional groups to the aromatic ring sterically preclude binding.

The catalytic versatility of Sad suggests several potential translational applications. Its reactivity toward aldehydes positions it as a promising candidate for the biocatalytic synthesis of steroid therapeutics, providing a more sustainable alternative to traditional chemical methods. Additionally, Sad exhibits bidirectional activity, catalyzing both the oxidation and reduction of steroid side chain aldehydes. This versatility could facilitate downstream applications, where these reactive aldehydes participate in further chemical transformations, potentially leading to novel steroids with medicinal properties. The capacity of Sad to process steroidal aldehydes points to potential bioremediation applications where the conversion of reactive, toxic aldehyde groups into less harmful compounds could reduce environmental contamination.

Beyond terrestrial soil bacteria, some additional microbial contexts may encode Sad-like aldehyde dehydrogenases with potential ecological and metabolic significance. For example, steroid catabolic genes have been identified in marine sediments as well as bacteria associated with marine sponges.^2^ Wastewater treatment systems represent recognized hotspots for microbial exposure to steroidal pollutants, where aldehyde intermediates can form during biodegradation and may require detoxification, potentially involving Sad-like enzymes.^2^ For example, anaerobic cholesterol degrading Proteobacteria, Sterolibacterium denitrificans, was isolated from a wastewater treatment system and contains steroid side chain degrading genes and a Sad homologue.^18^ While modification of bile acids by vertebrate intestinal bacteria is well documented, there is currently no evidence that gut bacteria are able to catabolize steroid rings and their side chains.^2,19^ Similarly, although plant-associated microbiomes, particularly in the rhizosphere, likely encounter phytosterols and other plant-derived triterpenoids, whether microbes in these niches utilize these compounds as carbon sources and possess the genetic potential to express Sad-like enzymes remain to be explored.^2^ Further investigation across diverse microbial habitats will therefore be essential for elucidating the full ecological distribution of Sad and the relevant steroid degradation pathways.

## Conclusions

The crystal structure of Sad_Ctepid_ revealed an enlarged substrate-binding pocket relative to other aldehyde dehydrogenase family 3 enzymes, enabling efficient oxidation of diverse bile acid and cholesterol intermediates, as well as several non-steroidal aldehydes. We demonstrate that another aldehyde dehydrogenase, *Pp*BADH, can be engineered to utilize steroid substrates by introducing key substitutions that mimic the active site of Sad_Ctepid_. Sad and other aldehyde dehydrogenases therefore represent compelling candidates for biocatalytic applications, especially in the synthesis or detoxification of steroid intermediates. Further exploration of steroid aldehyde dehydrogenases across diverse microbial environments will enhance our understanding of microbial steroid catabolism and unlock new opportunities for environmental and industrial applications.

## Materials and Methods

### Chemicals

Restriction enzymes and Pfu polymerase were obtained from Thermo Scientific (Ottawa, ON). T4 DNA ligase was obtained from New England Biolabs (Pickering, ON). Ni^2+^-NTA Superflow resin was obtained from Qiagen (Mississauga, ON). Cholic acid, chenodeoxycholic acid, and deoxycholic acid were obtained from Alfa Aesar (Ottawa, ON) and Sigma-Aldrich (Oakville, ON). 4-Pregnen-3-one-20β-carboxaldehyde and 4-pregnen-3-one-20β-carboxylic acid were purchased from Steraloids Inc. (Newport, RI). Coenzyme A was obtained from BioShop Canada Inc. (Burlington, ON), and ATP was obtained from Bio Basic Inc. (Markham, ON). All other chemicals were obtained from Thermo Scientific or Sigma-Aldrich unless stated otherwise.

### Bacterial Strains and Plasmids

C. testosteroni KF-1 was obtained from the DSMZ-German Collection of Microorganisms and Cell Cultures (Braunschweig, Germany), E. coli BL21 LOBSTR was obtained from Kerafast Inc. (Boston, MA, USA), C. tepidiphilia YIM 730274 was obtained from Korean Collection for type cultures, and P. putida ATCC12633 was obtained from the American Type Culture Collection.^20^

### DNA Manipulation

DNA purification, digestion, and ligation were performed according to the standard protocols. The genes for *sad* and *badh* were amplified from the genomic DNA of C. testosteroni KF-1, C. tepidiphilia, or P. putida using touchdown PCR.^21^ The PCR products were inserted into pET28a (Novagen, Madison, WI, USA) by using NdeI and *Hin*dIII to produce His-tagged proteins. Site-directed mutagenesis of BADH was performed using a modified QuikChange method, with the primers listed in Table S2.^22^ All plasmids were cloned in E. coli DH5α (Invitrogen, Carlsbad, CA, USA) following transformation. The cloned genes and point mutations were validated by DNA sequencing at Laboratory Services, University of Guelph (Guelph, ON, Canada). The transformed plasmids were subsequently introduced into E. coli BL21 LOBSTR for expression.^20^

### Protein Expression, Purification, and Analysis

Recombinant E. coli cultures were grown in 4 L of LB medium containing kanamycin (50 μg/mL) at 37 °C. At mid-log phase (OD_600_ = 0.4–0.6), protein expression was induced with 1 mM isopropyl-β-d-thiogalactopyranoside (IPTG). Cultures were incubated for 24 h at 15 °C, and cells were harvested by centrifugation (9605*g*, 10 min). Pellets were resuspended in 20 mM HEPES buffer (pH 7.5) and lysed via French press (103,421 kPa). Lysates were clarified by centrifugation (39,191*g*, 20 min), and supernatants were filtered through a 0.45 μm membrane. The filtered lysate was incubated with Ni^2+^-NTA resin in 20 mM HEPES buffer (pH 7.5) supplemented with 20 mM imidazole (pH 8.0) for 1 h at 4 °C. The mixture was loaded onto a gravity column and washed with the same buffer, and His-tagged proteins were eluted using 150 mM imidazole (pH 8.0). Eluted proteins were concentrated to 0.5–1 mL using a stirred cell with a YM10 filter (Amicon) and dialyzed into 20 mM HEPES buffer (pH 7.5). Purified proteins were stored at −80 °C. Protein concentration was quantified using the Bradford assay with bovine serum albumin as a standard, and purity was assessed by SDS-PAGE followed by Coomassie blue staining.^23^

### Differential Scanning Fluorimetry

The melting temperature (*T*_m_) of wild-type BADH and its variants was determined using SYPRO Orange dye. Fluorescence signals were monitored on a StepOnePlus Real-Time PCR system (Applied Biosystems, Foster City, USA). Experiments were performed in triplicate under standardized conditions: 0.3 mg/mL protein in 20 mM HEPES buffer (pH 7.5) with 1× SYPRO Orange, as described previously.^24^

### Preparation of Aldehyde-Functionalized Bile Acids

3α,7α,12α-Trihydroxy-5β-cholan-22-al was synthesized via established protocols with an added retro-aldol cleavage step.^4,25^ A 10 mL reaction mixture containing 100 mM sodium HEPES buffer (pH 7.5), cholate (1.0 mM), CoA (1.0 mM), ATP (2.5 mM), magnesium sulfate (5.0 mM), and Rhodococcus jostii RHA1 acyl-CoA synthetase CasG (1.5 μM) was incubated overnight at 22 °C with gentle agitation.^26^ The resulting CoA-esterified side chain was dehydrogenated, hydrated, and aldolytically cleaved in 40 mL of 100 mM sodium HEPES buffer (pH 7.5) using R. jostii RHA1 acyl-CoA dehydrogenase CasC (0.5 μM) with potassium hexacyanoferrate (III) (300 μM) and C. testosteroni Shy-Sal (0.5 μM) incubated for 2.5 h at 22 °C.^4,27^ The reaction was quenched by acidification to pH 4.0 with HCl, filtered through 0.45 μm and YM10 membranes, and purified via a HyperSep C18 column (2.8 mL bed volume, 500 mg; Thermo Scientific) equilibrated with 30% acetonitrile in 50 mM sodium phosphate buffer (pH 5.3). CoA esters were eluted with 40% acetonitrile in the same buffer. After evaporating acetonitrile under air, the product underwent three chloroform-water (1:1, v/v) extractions to isolate nonpolar molecules. The final product was dissolved in acetonitrile and analyzed by liquid chromatography–mass spectrometry (LC–MS). Identical protocols using deoxycholate or chenodeoxycholate as starting material were performed to synthesize 3α,7α-dihydroxy-5β-cholan-22-al and 3α,12α-dihydroxy-5β-cholan-22-al, respectively.

### Steady-State Kinetic Assays

Aldehyde dehydrogenase activity was examined in 200 μL reactions containing 1 mM NAD^+^ and varying concentrations of aldehyde substrates in 100 mM sodium HEPES buffer (pH 7.5) at 25 °C. The activity was determined by monitoring the increase in absorbance at 340 nm using the Agilent Biotek plate reader, corresponding to the reduction of NAD^+^ to NADH (with an extinction coefficient ε340 of 6.22 mM^–1^ cm^–1^). Data were fitted to the Michaelis–Menten equation using nonlinear regression with GraphPad Prism software.

### Protein Crystallization and X-ray Crystallography Data Acquisition

Crystallization conditions for Sad_Ctepid_ were screened by using the Pact Premier kit (Molecular Dimensions Inc.). Crystals suitable for data collection were obtained under optimized conditions: 40% (v/v) 2-methyl-2,4-pentanediol (MPD), 3% (w/v) PEG 8000, 0.1 M sodium cacodylate (pH 6.5), and 10% (v/v) glycerol. Crystallization was performed at 4 °C via the sitting-drop vapor diffusion method using 20 mg/mL Sad_Ctepid_. For cryoprotection, crystals were coated with Paratone-N prior to being flash-freezed in liquid nitrogen. X-ray diffraction data were collected at the Canadian Macromolecular Crystallography Facility (CMCF-BM; Canadian Light Source). Data sets were processed and scaled using XDS and XSCALE.^28^ Molecular replacement was conducted in Phaser with a ColabFold-generated model of the Sad_Ctepid_ homodimer as the search probe.^10^ Iterative refinement was performed using PHENIX.refine, coupled with manual model adjustments in Coot (Crystallographic Object-Oriented Toolkit).^29,30^ Structural figures were generated with PyMOL v2.0. Cavity analysis was performed using CavitOmiX v1.0 (Innophore GmbH, 2022). Hydrophobicity of identified cavities was analyzed with the hydrophobicity module in VASCo, and cavity dimensions were calculated using a modified LIGSITE algorithm.^31–34^

### Detection of Reverse Reaction Products by LC–MS

Reactions were performed in 5 mM ammonium acetate (pH 7.4) with 8 nmol Sad_Ctepid_, 50 μM 4-pregnen-3-one-20β-carboxylic acid, and 1 mM NADH. Liquid chromatography–mass spectrometry analyses were performed on Waters Acquity I-class UPLC coupled to a Synapt G2Si mass spectrometer at the Mass Spectrometry Facility of the Advanced Analysis Centre, University of Guelph. A C18 column (Agilent Poroshell 120, 50 mm × 4.6 mm 2.7 μm) was used for chromatographic separation with the following solvents: water with 15 mM ammonium formate (A) and acetonitrile (B). The mobile phase gradient was as follows: initial conditions were 2% B hold for 1 min then increasing to 98% B in 18 min followed by column wash at 98% B for 1 and 10 min re-equilibration. The flow rate was maintained at 0.4 mL/min. The mass spectrometer electrospray capillary voltage was maintained at 3.0 kV, the source temperature was 80 °C, cone gas 50 L/h, desolvation temperature was 600 °C, and desolvation gas flow was 1000 L/pressure with a nebulizing gas flow of 7 bar. The mass-to-charge ratio was scanned across the *m*/*z* range of 50–1500 *m*/*z* in the MS resolution mode using TOF MS. The instrument was externally calibrated with sodium iodide. Real-time lockmass correction was achieved by infusing leucine enkephalin at 10 μL/min through a lockspray probe and acquired every 30 s. The sample injection volume was 5 μL. Data analysis was performed using MassLynx Version 4.2 software (Waters).

## Supplementary Material



## Data Availability

The structure of the Sad_Ctepid_ complex has been deposited in the Protein Data Bank under accession code 9NXL. Raw crystallographic data have been deposited in the Zenodo repository under the filename Sad­(A.tepid)_9NXL (DOI: 10.5281/zenodo.15114451).

## Notes

Structure of cholate, bile acid catabolic pathway, sequence alignments, reverse catalysis reactions diagrams, LC–MS analysis, structural models, docking results, thermal shift assays, mutagenesis primers, and aldehyde dehydrogenase sequences used for alignment (PDF)
