# Supplementary material for: Sad from Proteobacteria is a Structurally Distinct ALDH3 Enzyme Specialized for the Oxidation of Steroidal Aldehydes
Source: Biochemistry. 2025 Aug 18;64(17):3735–44. doi: 10.1021/acs.biochem.5c00213 (PMC12410373; doi:10.1021/acs.biochem.5c00213)
Supplement: Supplementary file 1 [file bi5c00213_si_001.pdf]

Supporting Information

Article: *Sad from Proteobacteria is a Structurally Distinct ALDH3 Enzyme Specialized for the Oxidation of Steroidal Aldehydes*

Nicolas Rolfe, Dustin Myskiw, Matthew T Patton, Taylor JB Forrester, Matthew S Kimber, and Stephen YK Seah*

Department of Molecular and Cellular Biology, University of Guelph, Guelph, ON, Canada N1G 2W1

*Corresponding Author:

Dr. Stephen Seah, Email: sseah@uoguelph.ca; Tel: 1 (519) 824-4120 Ext: 56750;

Table of Contents

Figure S1. Structure of cholate S2

Figure S2. The bile acid side chain catabolic pathway in Proteobacteria..S2

Figure S3. Multiple sequence alignment of Sad and BADH homologs…….......S3 and S4

Figure S4. Sad reverse catalysis reactions for reduction of C_3 and C_5 side chain carboxylates..S4

Figure S5. LC-MS analysis of C_3 side chain carboxylate reduction by Sad_{Ctepid}..S5 and S6

Figure S6. Structure of $Sad_{A.tepid}$ with domains and secondary structures labelled……………………………………………………...S6

Figure S7. Planar steroid A ring in the Sad_{Ctepid} cavity..S7

Table S1. Thermal shift assay for wt *Pp*BADH and *Pp*BADH variants..S8

Table S2. Primers used for site-directed mutagenesis of *Pp*BADH……………………...S8

Table S3. List of aldehyde dehydrogenase sequences used in the multiple sequence alignment..S8

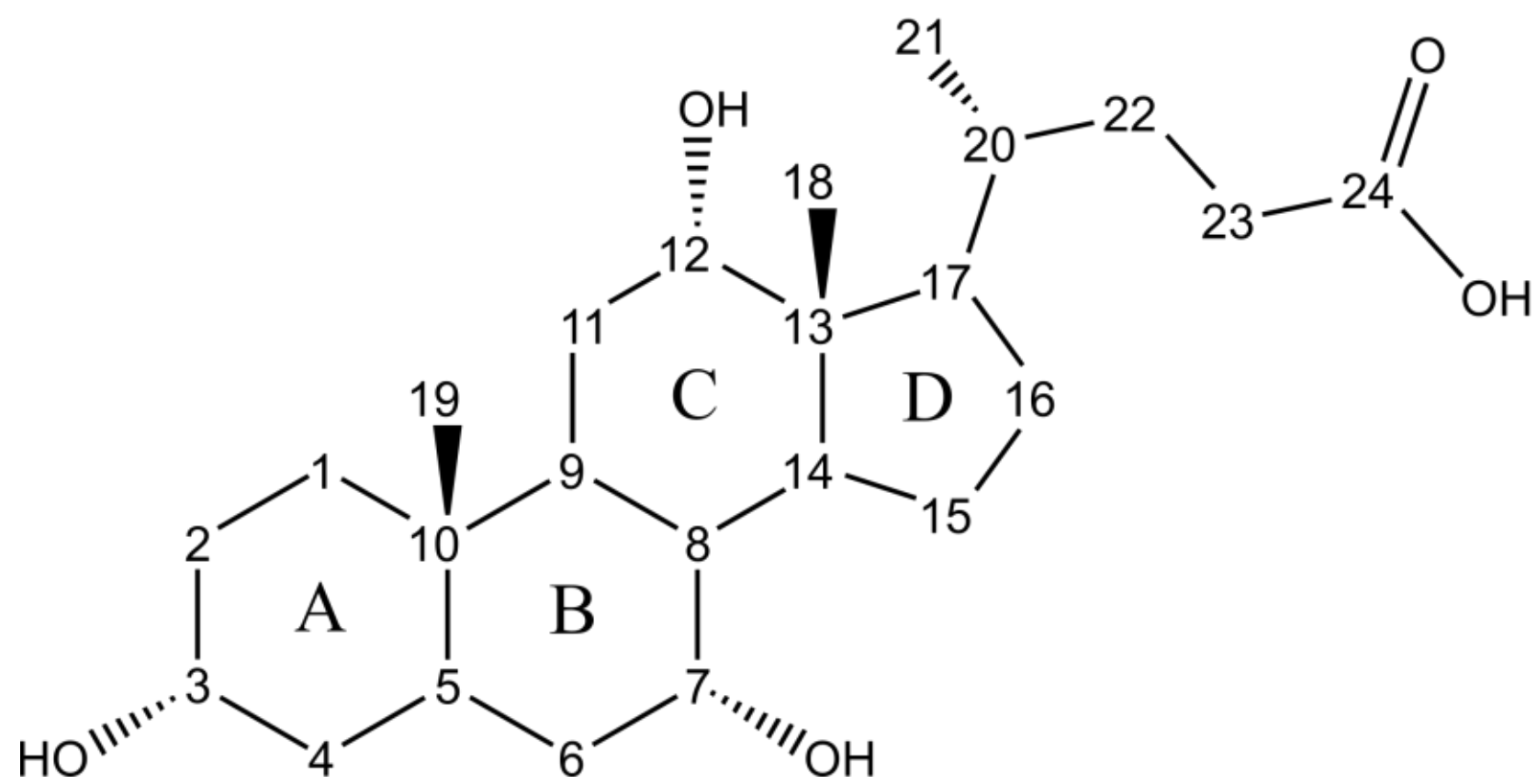

Figure S1. Structure of cholate. The chemical structure of the bile acid cholate is shown with carbon numbering (C1–C24) and labeled steroid rings (A–D).

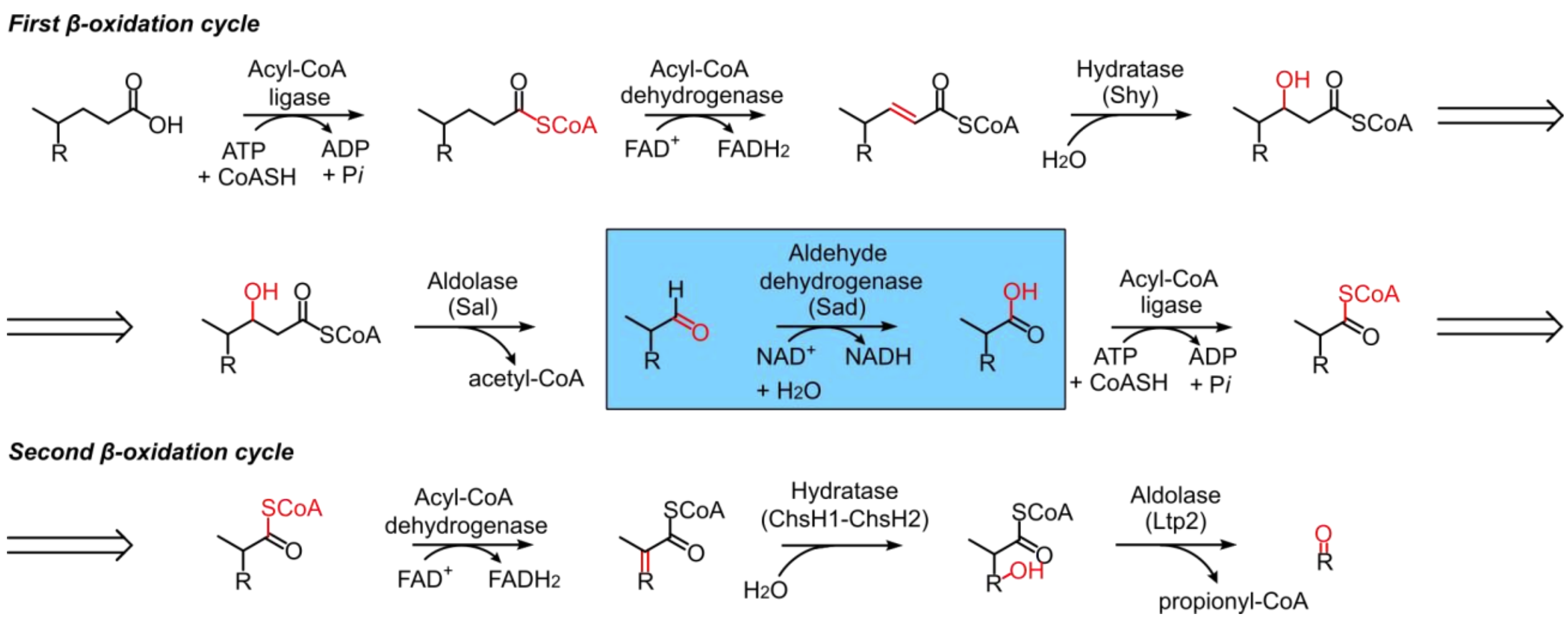

Figure S2. The bile acid side chain catabolic pathway in Proteobacteria. For clarity, the steroid nuclei are denoted as R groups. Complete degradation of the side chain requires two rounds of β-oxidative reactions. Transformed functional groups at each step are shown in red. The enzyme in this study, Sad, is boxed in blue.

```
                  1        10        20        30        40        50
SadCtest   .MTQTNSSIGIRQLLDMQKQAFIEEGAVSAEVRMQRLQQLIDMLVEHKNALCQA
SadCtepi   ..MPQTSPAPLRALLDAQRAAFLEDGGVPDTLRQARLQRVIDMLVEHHDALCAA
SadSdegr   MNANLRSIDEVGELLAAQRQAFDREGAVNLETRKRRLQAVIDLLVAEQGALVEA
BADHPputi  ..MNYLSPAKIDSLFSAQKAYFATRATADVGFRKQSLERLKEAVINNKEALYSA
BADHHsapi  .......MSKISEAVKRARAAFSSGRTRPLQFRIQQLEALQRLIQEQEQELVGA
BADHRnorv  .......MSSISDTVKRAREAFNSGKTRSLQFRIQQLEALQRMINENLKSISGA

                    60        70        80        90       100
SadCtest   MGEDFGGRPAVFSLANDILGSLASLKHARDHLKDWLGDSVRPSVKPFDMFGATA
SadCtepi   MEQDFGGRSRVFSLMNDVLGSLASLKHARDQLPNWTPREPRPSVAPFDSFGATA
SadSdegr   MDADFGGRHPGYGLMTDILGALASLKHAREHVDGWMQRDARPAFAPYDQLGAEA
BADHPputi  LAEDLGKPKDV.VDLAEIGAVLHEIDFALAHLDEWVAPVSVPSPD..IIAPSEC
BADHHsapi  LAADLHKNEWN.AYYEEVVYVLEEIEYMIQKLPEWAADEPVEKTP..QTQQDEL
BADHRnorv  LASDLGKNEWT.SYYEEVAHVLEELDTTIKELPDWAEDEPVAKTR..QTQQDDL

           110       120       130       140       150       160
SadCtest   WVKYQPKGVIGIIGTWNAPLFTLLSPLACAFAAGNRAVLKPSEIAPRTAQVLAE
SadCtepi   WVQYQPKGVVGIVGTWNAPLFTLFSPLASVLAAGNRAVLKPSELTPRTAEAVAQ
SadSdegr   WVQYQPKGSVGIIGTWNAPLFTLLSPLAGVLAAGNRAILKPSEVTANTAAVLAA
BADHPputi  YVVQEPYGVTYIIGPFNYPVNLTLTPLIGAIIGGNTCIIKPSETTPETSAVIEK
BADHHsapi  YIHSEPLGVVLVIGTWNYPFNLTIQPMVGAIAAGNSVVLKPSELSENMASLLAT
BADHRnorv  YIHSEPLGVVLVIGAWNYPFNLTIQPMVGAVAAGNAVILKPSEVSGHMADLLAT

                     170       180       190       200       210
SadCtest   AVAKRFDPQVLAVVPGGPEVAAAFAEQPWNHLVFTGSTSVGKLIMAAAARNLVP
SadCtepi   AVAAFFAPEELAVVTGGPDVGAEFAAQPWDHLVFTGSTAVGKRVMQAAAENLVP
SadSdegr   AIGERFDPLELAVVTGGVEVGQGFASQPFDHLVFTGSTAIGRDVMRRAAENLVP
BADHPputi  IIAEAFAPEYVAVIQGGRDENSHLLSLPFDFIFFTGSPNVGKVVMQAAAKHLTP
BADHHsapi  IIPQYLDKDLYPVINGGVPETTELLKERFDHILYTGSTGVGKIIMTAAAKHLTP
BADHRnorv  LIPQYMDQNLYLVVKGGVPETTELLKERFDHIMYTGSTAVGKIVMAAAAKHLTP

              220       230       240       250       260
SadCtest   VTLELGGKSPVLVSDTADIAHAAERIAVGKSLNSGQLCVSPDEVWVHESRLQAL
SadCtepi   VTLELGGKSPVLLSPGADLRNAAERIAVGKSSNSGQLCVAPDVVWVHESQAQAF
SadSdegr   VTLELGGKSPVIVGRSADIADAARRLAIAKLTNGGQICVSPDLVYVPVEHKQAF
BADHPputi  VVLELGGKCPLIVLPDADLDQTVNQLMFGKFINSGQTCIAPDYLYVHYSVKDAL
BADHHsapi  VTLELGGKSPCYVDKNCDLDVACRRIAWGKFMNSGQTCVAPDYILCDPSIQNQI
BADHRnorv  VTLELGGKSPCYVDKDCDLDVACRRIAWGKFMNSGQTCVAPDYILCDPSIQNQI

         270       280       290       300       310       320
SadCtest   AEGIAAQYQSLYPS.VSGNADMTPVINERHHARVKAYVDDAKARGLSVLEVGA.
SadCtepi   VEALDEVWRGLFPT.IAGNRDVVPVVNERHFARVESYVADAAERGARVLLVGGW
SadSdegr   IEAFRAAVTALYPS.ANDNPDLVAVVNERHLQRIDRYLADAVEQGAEVLHCPGE
BADHPputi  LERLVERVKTEL...PEINSTGKLVTERQVQRLVSLLEATQG...QVLVGSQA
BADHHsapi  VEKLKKSLKEFYGEDAKKSRDYGRIISARHFQRVMGLIEGQ.....KVAYGGTG
BADHRnorv  VEKLKKSLKDFYGEDAKQSRDYGRIINDRHFQRVKGLIDNQ.....KVAHGGTW

                 330       340       350       360       370
SadCtest   ..AQAGSDRRLPLALVINPPADAEISRHEIFGPALVLRSFHNIRDAVAAIN.AG
SadCtepi   PSAQGTAERRMPLRVVMNPPADSEIARHEIFGPALVLNTFRELPQAVAAIN.AG
SadSdegr   ..APVASSRKRPLSLVVNPPLHSRVMEEELFGPIMVLLTYSDLPSVVAEIN.AR
BADHPputi  ....DVSKRALSATVVDGVEWNDPLMSEELFGPILPVLEFDSVRTAIDQVNKHH
BADHHsapi  ....DAATRYIAPTILTDVDPQSPVMQEEIFGPVLPIVCVRSLEEAIQFIN.QR
BADHRnorv  ....DQSSRYIAPTILVDVDPQSPVMQEEIFGPVMPIVCVRSLEEAIQFIN.QR

                 380       390       400       410       420
SadCtest   ERPLALYYFGSDETEQGWVLDNTLSGGVSINDVVMHPALEDAPFGGVGASGMGH
SadCtepi   ERPLALYYFGSDEAEQRWVLEHTLSGGVSINDVCMHPALHDAPFGGIGASGMGR
SadSdegr   PRPLALYYFGQNPAEQDYVLSHTLSGGVTINDAMLHPAMHDAPFGGVGASGMGH
BADHPputi  PKPLAVYVFGKDMDVAKGIINQIQSGDAQVNGVMLHAFSPYLPFGGIGASGMGE
BADHHsapi  EKPLALYMFSSNDKVIKKMIAETSSGGVAANDVIVHITLHSLPFGGVGNSGMGS
BADHRnorv  EKPLALYVFSNNEKVIKKMIAETSSGGVTANDVIVHITVPTLPFGGVGNSGMGA

             430       440       450       460       470
SadCtest   YHGREGFLEFSHARSVYKAGSHDPRREWGMLPPYNEQFAQMLQATVTP.
SadCtepi   YHGREGFLEFSHARSVYQAGSHDPRREWGMLPPYGEQFEAMLRASVTR.
SadSdegr   YHGREGFCQFSHARTVFKAPAYDPRGEWGMLPPYGEQFLMAMQAQVTAD
BADHPputi  YHGHFSYLTFTHKKSVRIVP.............................
BADHHsapi  YHGKKSFETFSHRRSCLVRPLMNDEGLKVRYPPSPAKMTQH........
BADHRnorv  YHGKKSFETFSHRRSCLVKSLLNEEAHKARYPPSPAKMPRH........
```

Figure S3. Multiple sequence alignment of Sad and BADH homologs. Aligned sequences include: *Comamonas testosteroni* Sad (SadCtest), *Caldimonas tepidiphila* Sad (SadCtepi), *Stutzerimonas degradans* Sad (SadSdegr), *Pseudomonas putida* BADH (BADHPputi), *Homo sapiens* BADH (BADHHsapi), and *Rattus norvegicus* BADH (BADHRnorv). The nucleophilic cysteine is highlighted in blue. Residues highlighted in cyan are proposed to function as a glutamate residue that activates a water molecule that hydrolyzes the thioacyl intermediate, an asparagine that provides the oxyanion hole, and an additional glutamate residue that was thought to function in hydride transfer to the nicotinamide cofactor. Other residues that are fully conserved are shown in red, while those that are partially conserved are shown in yellow. Accession numbers of sequences are in Table S3.

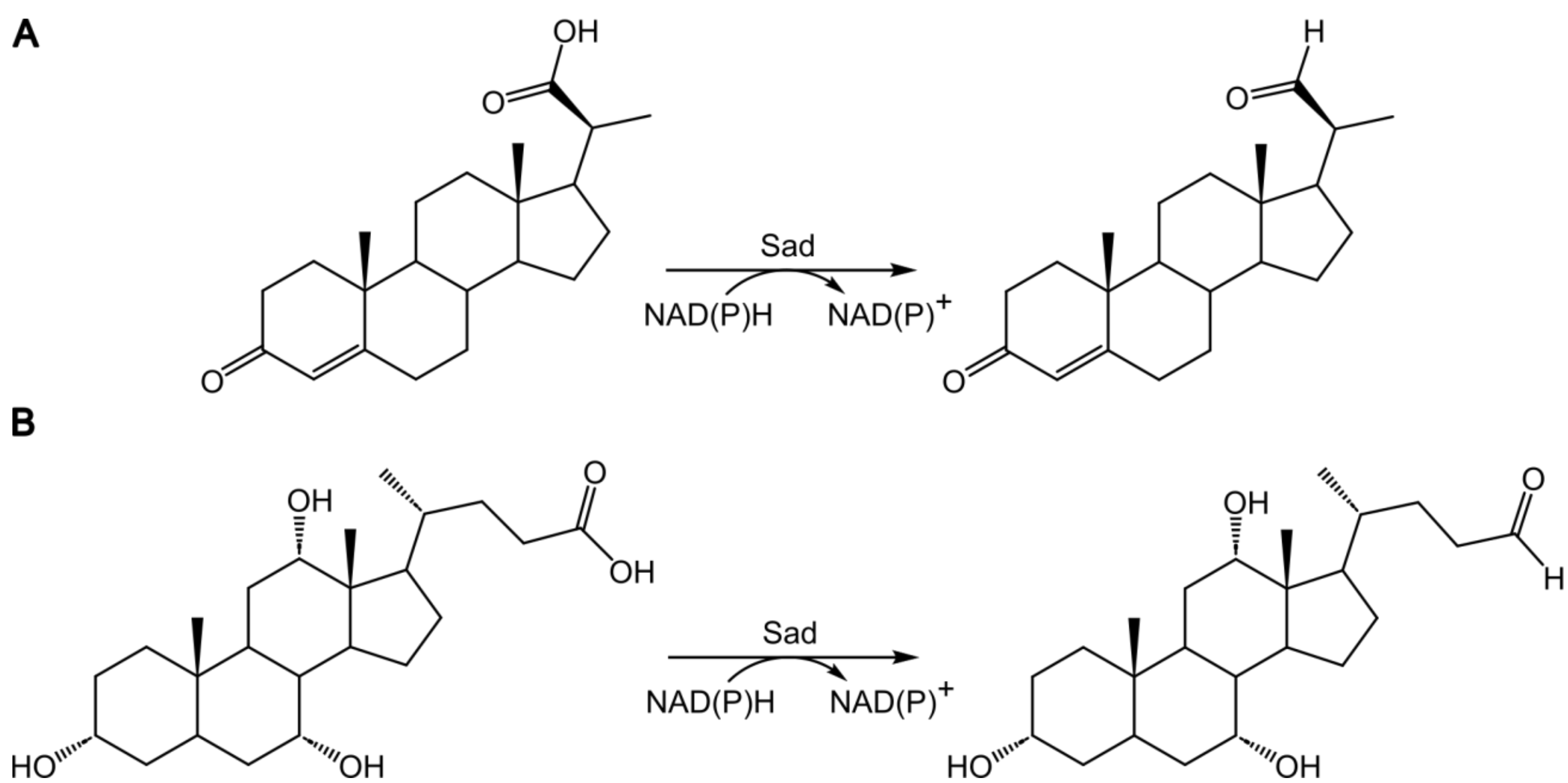

Figure S4. Sad reverse catalysis reactions for reduction of C_3 and C_5 side chain carboxylates. (A) Reduction of 4-pregnen-3-one-20β-carboxylic acid to 4-pregnen-3-one-20β-carboxaldehyde and (B) reduction of cholic acid to cholic aldehyde by Sad with a protonated nicotinamide cofactor.

A

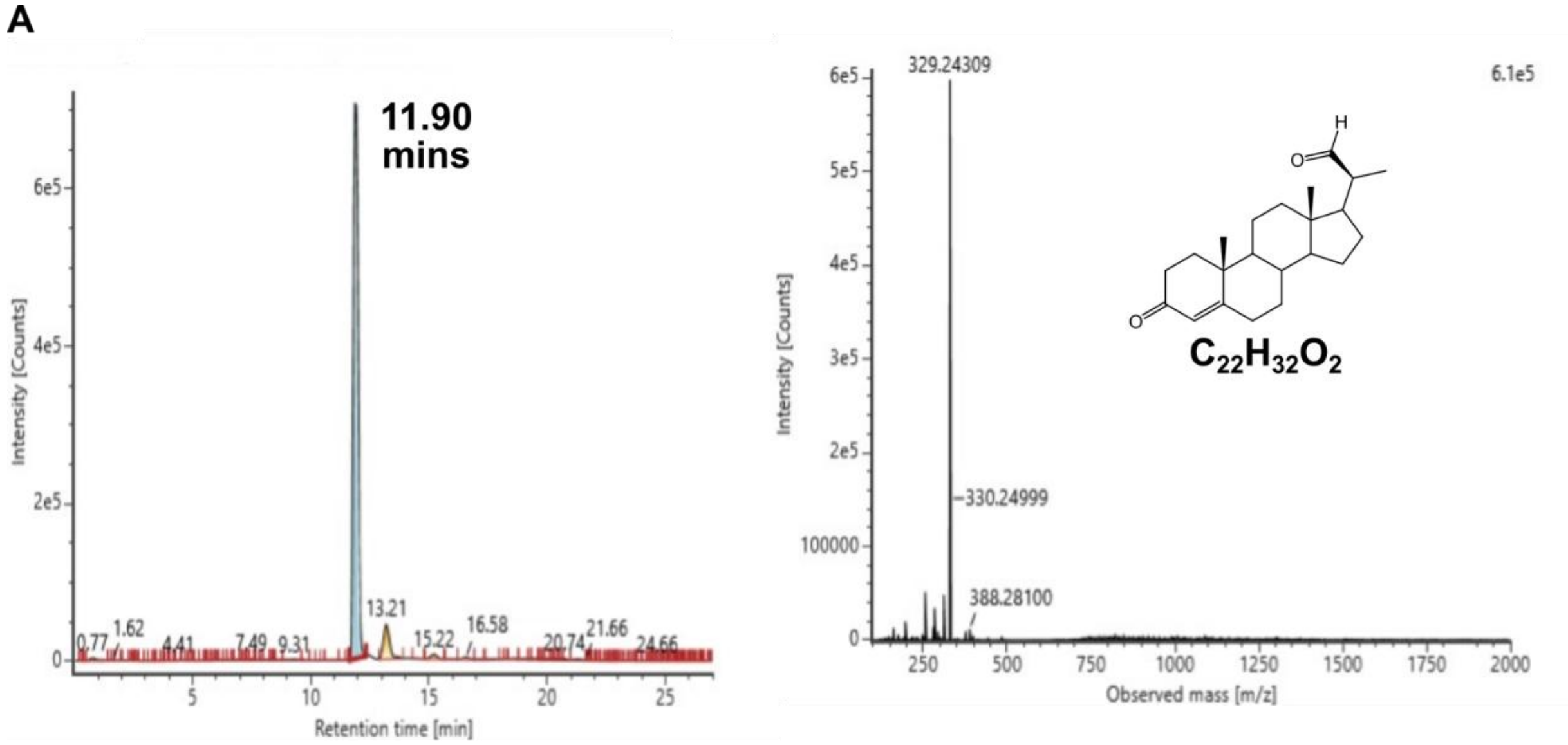

11.90
mins
Intensity [Counts]
Retention time [min]
0.77
1.62
4.41
7.49
9.31
13.21
15.22
16.58
20.74
21.66
24.66
329.24309
6.1e5
330.24999
388.28100
Observed mass [m/z]
$C_{22}H_{32}O_2$

B

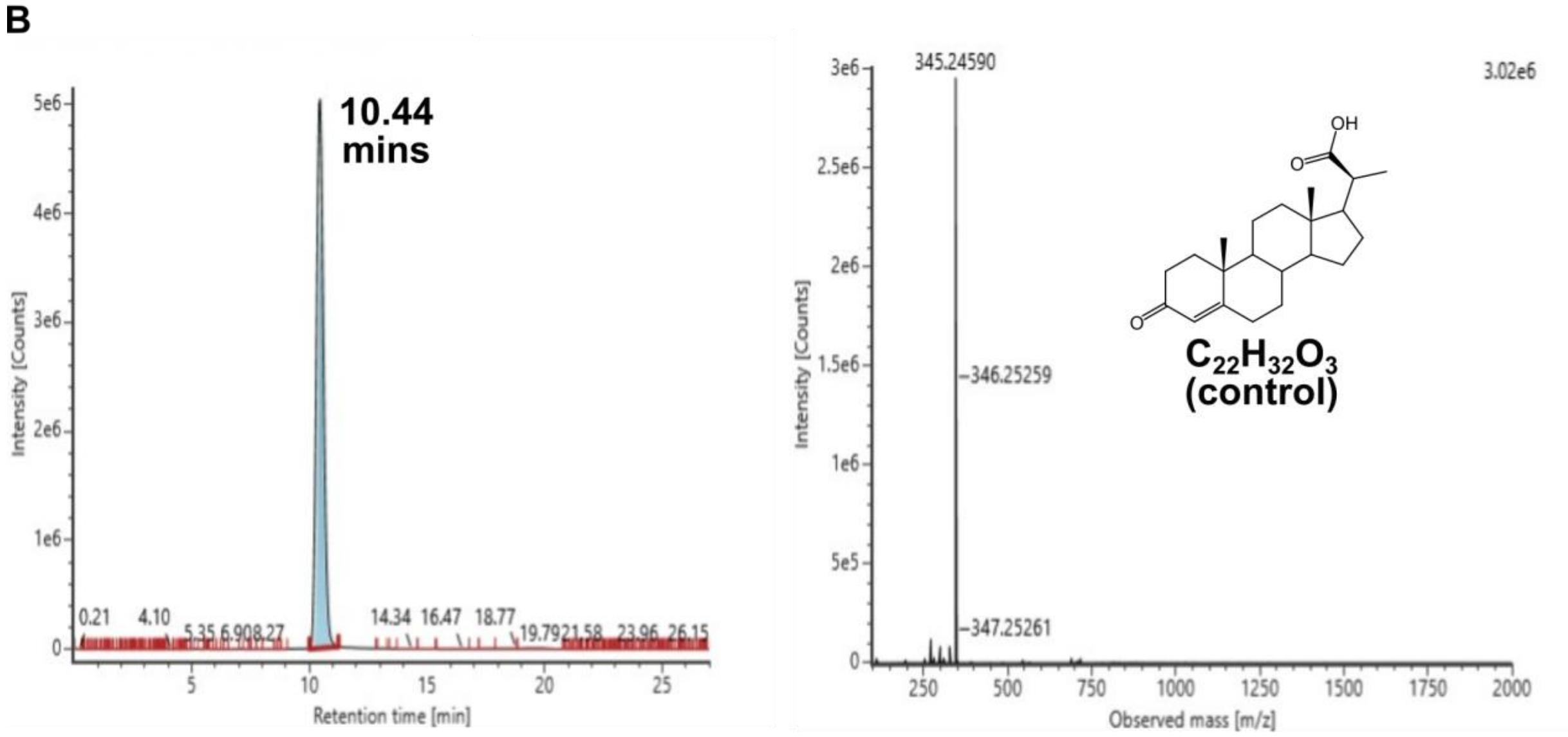

10.44
mins
Intensity [Counts]
Retention time [min]
0.21
4.10
5.35
6.908.27
14.34
16.47
18.77
19.79
21.58
23.96
26.15
345.24590
3.02e6
346.25259
347.25261
Observed mass [m/z]
$C_{22}H_{32}O_3$
(control)

C

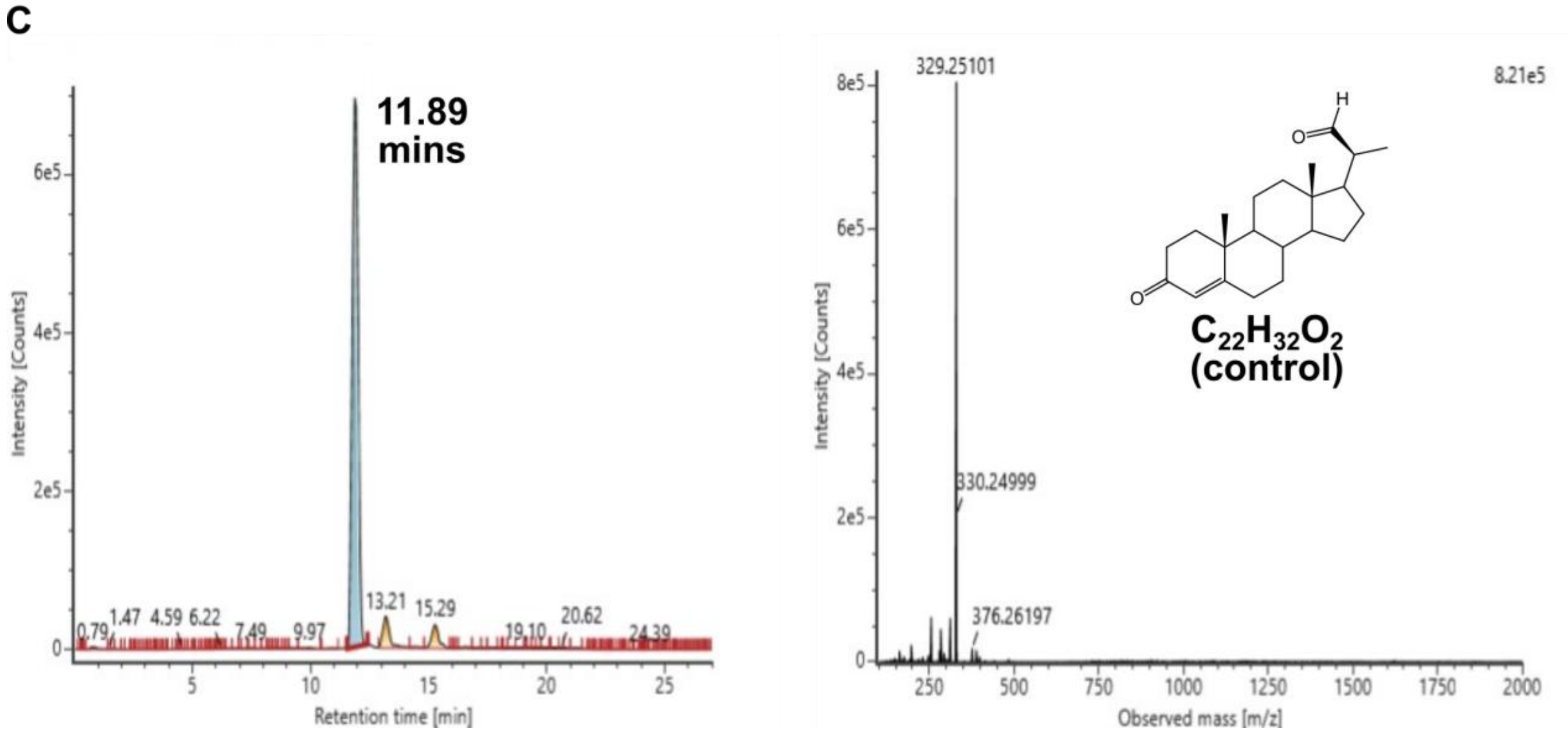

11.89
mins
Intensity [Counts]
Retention time [min]
0.79
1.47
4.59
6.22
7.49
9.97
13.21
15.29
19.10
20.62
24.39
329.25101
8.21e5
330.24999
376.26197
Observed mass [m/z]
$C_{22}H_{32}O_2$
(control)

Figure S5. LC-MS analysis of C_3 side chain carboxylate reduction by Sad_{Ctepid}. (A) Product from reduction of 4-pregnen-3-one-20β-carboxylic acid (C_3 side chain carboxylate) by Sad_{Ctepid}. A peak with a retention time of 11.9 minutes and an m/z of 329, is consistent with 4-pregnen-3-one-20β-carboxaldehyde. (B) Control of 4-pregnen-3-one-20β-carboxylic acid. A single peak was observed with a retention time of 10.4 minutes and an expected m/z of 345. No signal for the aldehyde was detected in this sample. (C) Control of 4-pregnen-3-one-20β-carboxaldehyde. A peak was observed at 11.9 minutes with an expected m/z of 329, matching the retention time and mass of the product from the reduction of 4-pregnen-3-one-20β-carboxylic acid by Sad_{Ctepid}.

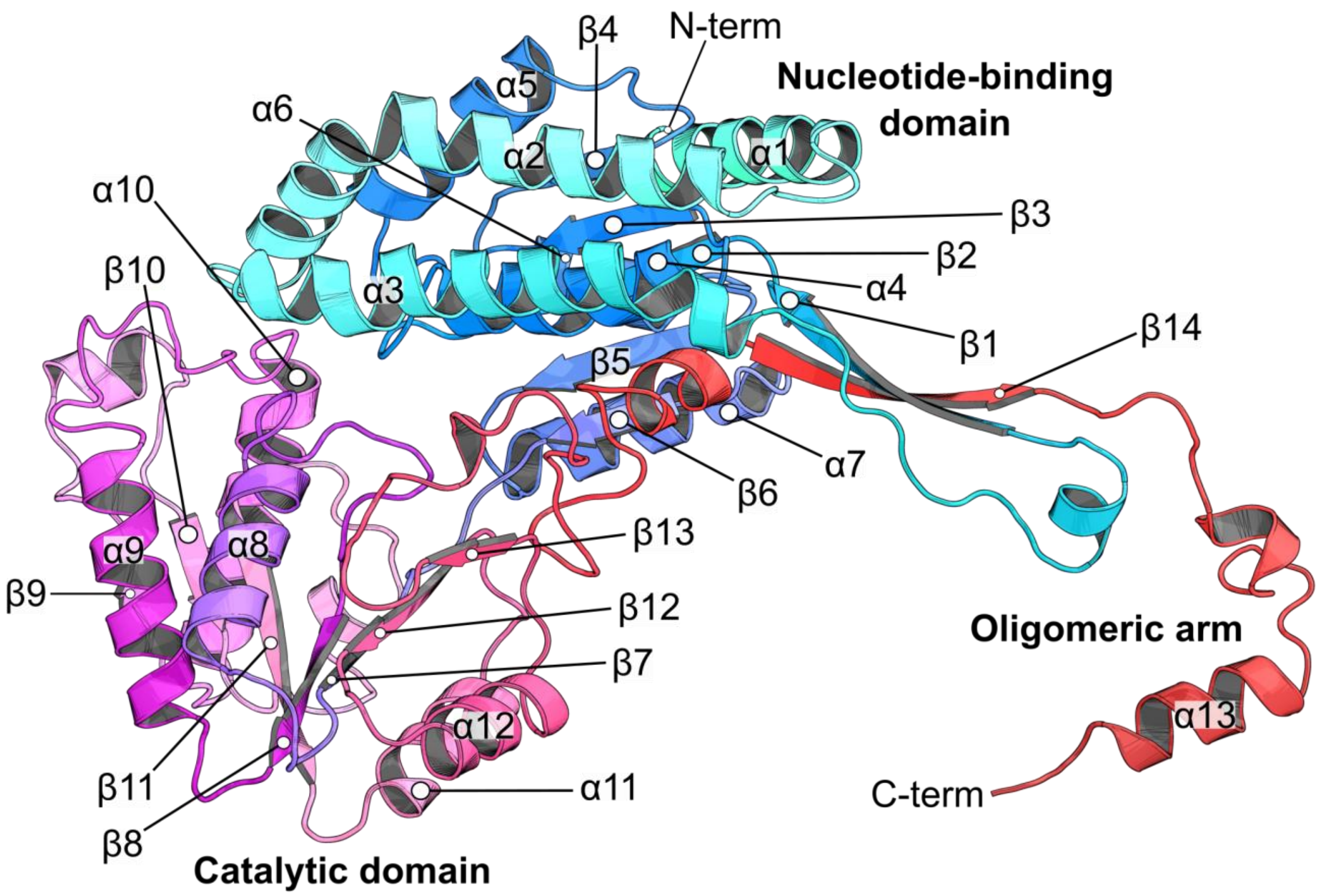

Figure S6. Structure of $Sad_{A.tepid}$ with domains and secondary structures labelled. The structure is coloured from N-terminus to C-terminus in the following order: cyan, blue, purple, pink, then red.

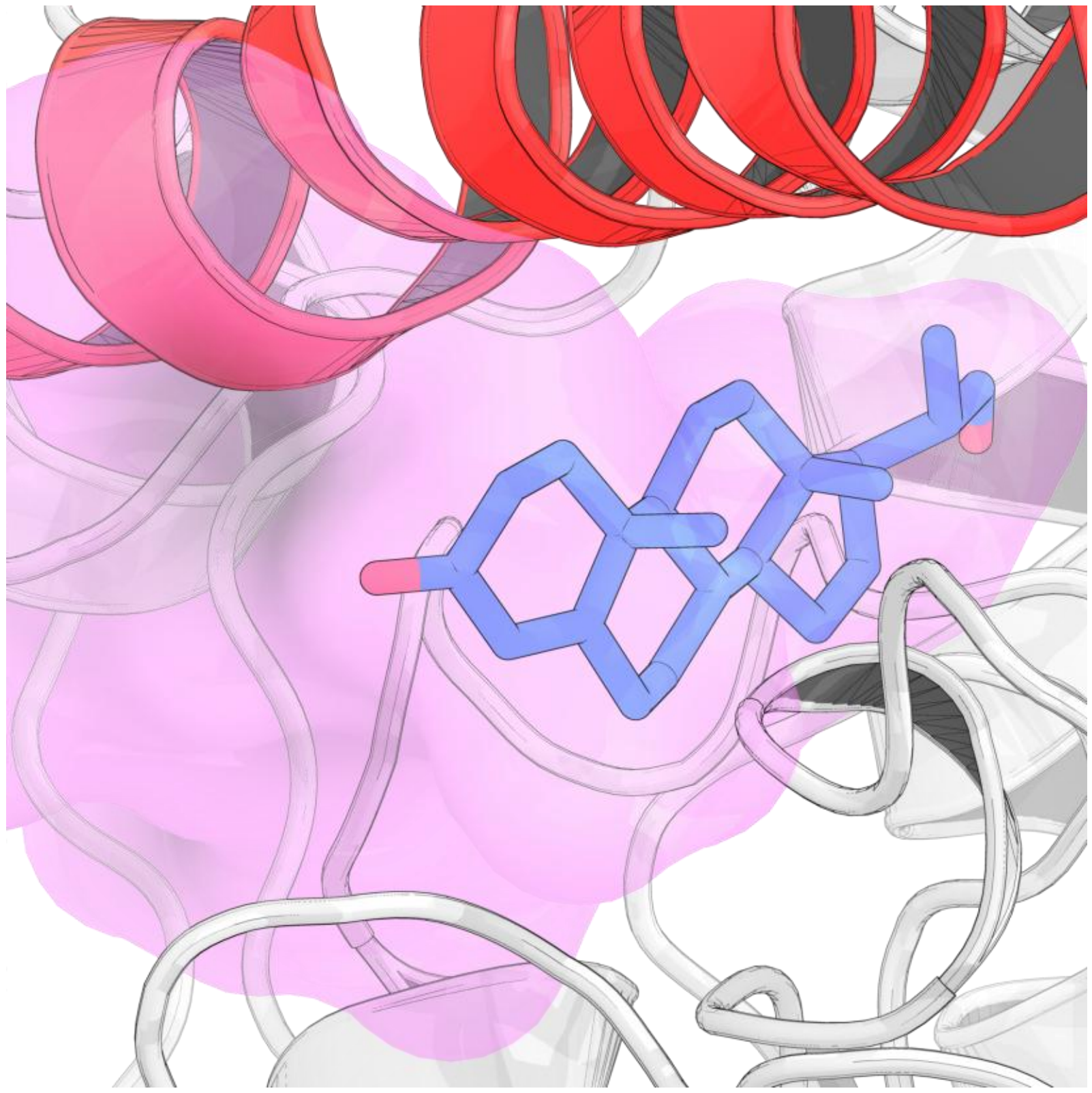

Figure S7. Planar steroid A ring in the Sad$_{Ctepid}$ cavity. Docking of the 4-pregnen-3-one-20β-carboxaldehyde (blue) showing that the planar A ring of the steroid fits inside the large cavity (violet) without steric clashes.

Table S1: Thermal shift assay for wt *Pp*BADH and *Pp*BADH variants.

***Pp*BADH Varient**	**Tm (°C)**	**$\|\Delta Tm\|_{wt\text{-}varient}$ (°C)**
wt	47.9 ± 0.818	-
E75S	47.9 ± 0.0817	0
Y121A	46.8 ± 0.818	1.1
H74A	47.2 ± 0.424	0.7
A399P	48.9 ± 0	1
I250V	47.5 ± 0.424	0.4
F400A	48.5 ± 0.519	0.6
L125T	43.8 ± 0	4.1
L125T;F400A	44.5 ± 0.920	3.4

Table S2: Primers used for site-directed mutagenesis of *Pp*BADH. Mutated codons are highlighted in red.

***Pp*BADH primer**	**Primer sequence**
L125T	F: CCAGTTAAC**ACG**ACTCTCACCCCGCTGATTGGTGCGATAATCG<br>R: GGTGAGAGT**CGT**GTTAACTGGGTAGTTGAAGGGGCCGATGATATAGG
F400A	F: ATGCG**GCC**TCGCCATACCTCCCGTTCGGGGGAATCG<br>R: GGCGA**GGC**CGCATGGAGCATTACGCCGTTCACCTGC
I250V	F: CAAACCTGC**GTT**GCACCGGATTACCTCTATGTGCATTACAGTGTCAAAGATGCAC<br>R: CGGTGC**AAC**GCAGGTTTGACCACTGTTTATGAATTTACCGAACATCAATTGATTGA
Y121A	F: CCCTTCAAC**GCC**CCAGTTAACCTGACTCTCACCCCGCTGATTGG<br>R: TTAACTGG**GGC**GTTGAAGGGGCCGATGATATAGGTGACACCGTACGG
H74A	F: GCAGTATTG**GCC**GAAATTGATTTTGCGCTTGCTCACCTTGATGAATGG<br>R: AAAATCAATTTC**GGC**CAATACTGCACCGATCTCTGCCAGGTCGAC
E75S	F: GTATTGCAC**TCA**ATTGATTTTGCGCTTGCTCACCTTGATGAATGGGTCG<br>R: CGCAAAATCAAT**TGA**GTGCAATACTGCACCGATCTCTGC
A399P	F: GCTCCAT**CCG**GCCTCGCCATACCTCCCGTTCGGG<br>R: GAGGC**CGG**ATGGAGCATTACGCCGTTCACCTGCG

Table S3: List of aldehyde dehydrogenase sequences used in the multiple sequence alignment.

**Species**	**Abbreviated name**	**Refseq accession number/GeneBank ID**
Comamonas testosteroni	Sad_{Ctest}	WP_043005812.1
Caldimonas tepidiphila	Sad_{Ctepi}	WP_119155807.1
Stutzerimonas degradans	Sdegr	EKM95645.1
Pseudomonas putida	Pputi	WP_016501743.1
Homo sapiens	Hsapi	NP_000682.3
Rattus norvegicus	Rnorv	NP_114178.1
